# CircSMEK1 Suppresses HCC via the hnRNPK‐IGF2‐AKT Axis: A Diagnostic Biomarker and Therapeutic Target

**DOI:** 10.1002/advs.202505267

**Published:** 2025-10-17

**Authors:** Peilan Guo, Xiaomeng Jia, Shenghong Wang, Xinyu Li, Yajing Liu, Lisen Lin, Zhengkun Wang, Fujun Liu, Slawomir Wolczynski, Nafis Rahman, Jie Gao, Xuguang Du, Suk‐Ying Tsang, Jiali Liu, Wei Song, Xiangdong Li

**Affiliations:** ^1^ State Key Laboratory of Animal Biotech Breeding College of Biological Sciences China Agricultural University Beijing 100193 China; ^2^ Shandong Provincial Hospital Affiliated to Shandong First Medical University Shandong 250021 China; ^3^ Department of Reproduction and Gynecological Endocrinology Medical University of Bialystok Bialystok 37801 Poland; ^4^ Department of Physiology Institute of Biomedicine University of Turku Turku 20520 Finland; ^5^ Yantai Yuhuangding Hospital Affiliated to Qingdao University Shandong 264000 China; ^6^ School of Life Sciences The Chinese University of Hong Kong Hong Kong SAR China

**Keywords:** circRNA, HCC, IGF2, MASLD, serum biomarker

## Abstract

The mechanism underlying metabolic dysfunction‐associated steatohepatitis (MASH) to hepatocellular carcinoma (HCC) is elusive, and whether circRNA can serve as biomarker or therapeutic target for MASH/HCC needs to be systematically explored. Integrative transcriptomic analysis of circRNA from MASH and HCC were performed. Multi‐cohort analyses of serum and tissues from MASH and HCC patients (*n* = 206) were conducted. Mechanisms are explored via RNA‐protein interaction assays, CRISPR‐mediated knockdown, and xenograft/PiggyBac‐mediated mice models. circSMEK1 is significantly decreased in MASH/HCC tissues and serum, correlating with tumor size, vascular invasion, and overall survival. Mechanistically, nuclear circSMEK1 binds hnRNPK, promoting its ubiquitin‐mediated degradation, suppressing IGF2 transcription and PI3K/AKT signaling. Loss of circSMEK1 elevated autocrine IGF2 in HCC promoting tumor growth, also activated AKT in cancer‐associated fibroblasts through paracrine, fostering an immunosuppressive microenvironment. SF3B4 overexpression drove circSMEK1 depletion in HCC. In murine models, circSMEK1 restoration inhibited tumor growth and metastasis. circSMEK1 is a tumor‐suppressor in MASH/HCC through the hnRNPK‐IGF2‐AKT axis. The serum level of circSMEK1 has non‐invasive diagnostic value for HCC (AUC = 0.790), as well as potential diagnostic utility for early HCC or high‐risk MASH, owing to its key role in bridging MASH to HCC progression. Restoring of circSMEK1, alone or combined with IGF2 inhibitors, proposing a novel therapeutic strategy for HCC.

## Graphical abstract

CircSMEK1 is downregulated in MASH/HCC and predicts poor prognosis. It suppresses tumor progression by promoting hnRNPK ubiquitination and inhibiting the IGF2/PI3K/AKT axis, while its loss activates immunosuppressive cancer‐associated fibroblasts. Serum circSMEK1 serves as a non‐invasive diagnostic biomarker, and its restoration potently inhibits HCC growth and metastasis in vivo.

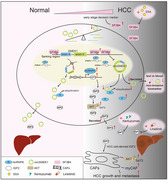

## 1 Introduction

Liver cancer remains a major global health burden, ranking as the sixth most common cancer and the third leading cause of cancer mortality worldwide.^[^ ^1^ ^]^ Hepatocellular carcinoma (HCC) accounts for ≈80%–90% of primary liver cancers, with China accounting for ≈50% of global cases.^[^ ^2^ ^]^ While hepatitis B virus (HBV) has historically driven HCC incidence in China (≈85% of HCC cases),^[^ ^3^ ^]^ metabolic dysfunction‐associated steatotic liver disease (MASLD, formerly NAFLD – non‐alcoholic fatty liver disease), a major HCC driver in Western countries,^[^ ^4^ ^]^ is emerging as a critical etiology, with current rates reaching 32.9%.^[^ ^5^ ^]^ MASLD affects ≈25% of the global population.^[^ ^6^ ^]^ Although vaccination has reduced HBV‐related HCC risk in China over the decades,^[^ ^7^ ^]^ the incidence of MASLD/ MASH (metabolic dysfunction‐associated steatohepatitis, formerly NASH‐non‐alcoholic steatohepatitis)‐ associated HCC (MASH‐HCC) is estimated to rise by 82% from 2016 to 2030,^[^ ^8^ ^]^ emerging as one of the major HCC causes. Alarmingly, over 38% of MASH progress directly to HCC without cirrhosis, indicating a unique pathogenesis from the traditional “hepatitis‐cirrhosis‐HCC” pathway.^[^ ^9^ ^]^ This suggests the shared dysregulation between MASH and HCC, underscoring the need to identify the common biomarkers and mechanisms.

Recently, new systemic treatments, particularly immune‐checkpoint inhibitors (ICIs) (atezolizumab/bevacizumab or durvalumab/tremelimumab), have significantly improved the outcomes of the advanced HCC and are the standard first‐line treatments.^[^ ^10^ ^]^ MASH‐HCC is also treated using the same approach. Currently, tumors are classified as inflamed or non‐inflamed based on the immune microenvironment. Inflamed HCC patients (≈30% cases) typically have a favorable prognosis and respond well to ICIs. Conversely, recent three Phase‐III trials involving over 1600 patients with advanced HCC showed that ICIs failed to improve overall survival (OS) in non‐viral HCC patients, MASH‐HCC patients treated with ICIs showed even reduced OS in two cohorts,^[^ ^11^ ^]^ highlighting the need for novel biomarker‐based patient stratification to optimize therapy response.

Circular RNAs (circRNAs), stable covalently closed transcripts, are increasingly implicated in HCC progression.^[^ ^12^ ^]^ Despite differences in etiology, MASH‐HCC and virus‐related HCC share common consequences leading to hepatocyte damage and carcinogenesis. Although several circRNA expression profiles in MASLD/MASH have been reported (in mice and human cell lines),^[^ ^13^ ^]^ their clinical relevance and mechanistic roles in human MASH‐HCC remain unexplored.

Here, we hypothesize that circRNAs dysregulated in both MASH and HCC may serve as shared biomarkers and therapeutic targets. By integrating multi‐cohort transcriptomic analyses and functional studies, we aim to: 1) identify circRNAs critical to MASH‐HCC progression, 2) elucidate their mechanisms in HCC pathogenesis, and 3) evaluate their diagnostic and therapeutic potential. Our findings may bridge a critical gap in understanding MASH‐HCC and pave the way for precision strategies.

## 2 Results

### 2.1 circSMEK1 is Downregulated in MASH/HCC and has a Prognostic Serum‐Biomarker Potential

**Figure 1:**
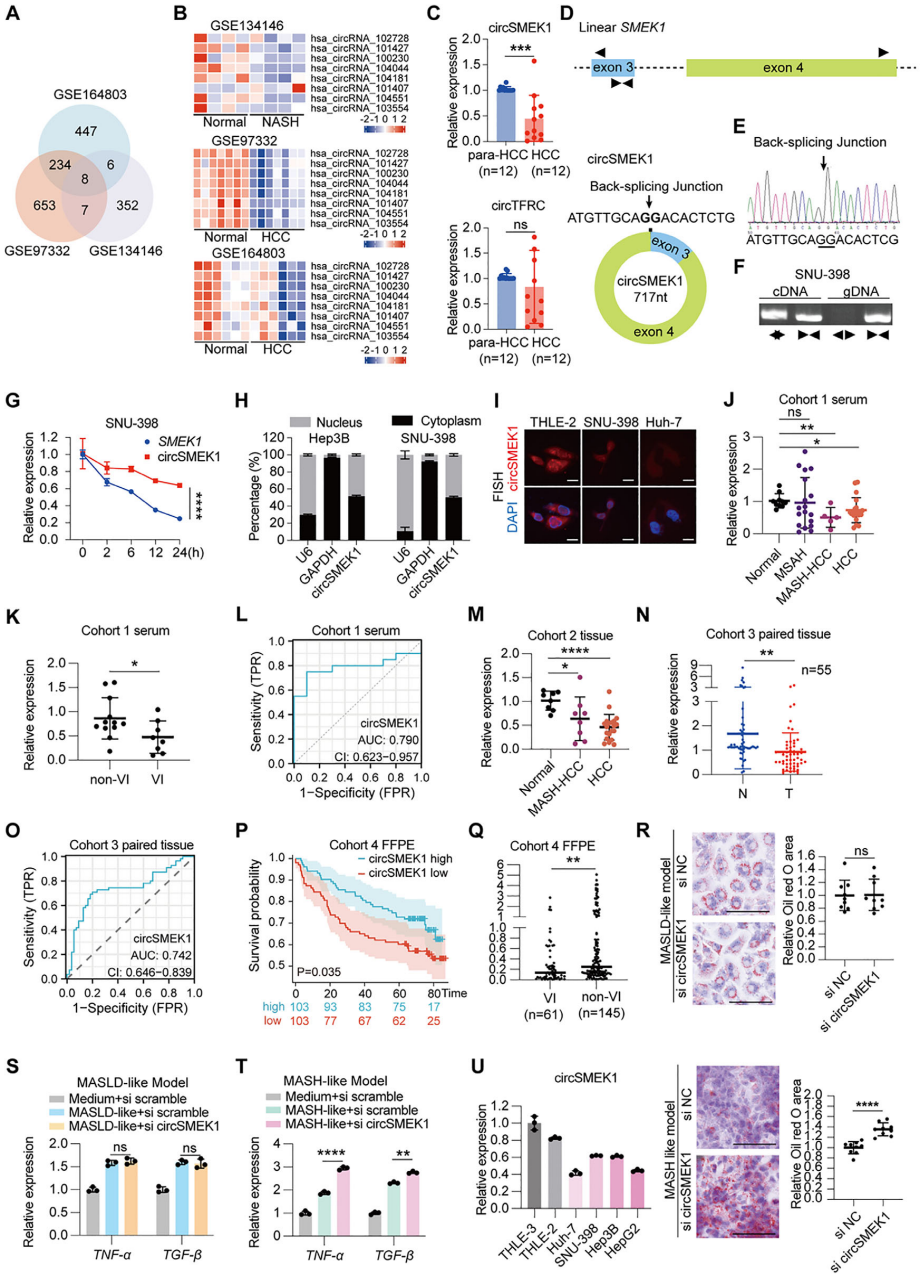
Characterization and expression of circSMEK1 in MASH and HCC. A) Analysis of circRNA expression profiles from MASH and HCC datasets. B) Heatmap of the top 8 differentially expressed circRNAs. C) Relative expression of circSMEK1 and circTFRC in paired peritumoral (Para‐HCC) and HCC tissues (*n* = 12 pairs). D) Schematic of circSMEK1 derived from exons 3–4 of SMEK1 via back‐splicing. E) Sanger sequencing validation of the back‐splicing junction (BSJ). F) PCR product through gDNA and cDNA using convergent or divergent primers G) Stability of circSMEK1 and linear SMEK1 mRNA in actinomycin D‐treated cells (*n* = 3). H) Nuclear/cytoplasmic distribution of circSMEK1 in HCC cells (*n* = 3). U6 and GAPDH served as nuclear and cytoplasmic controls, respectively. I) RNA fluorescence in situ hybridization (FISH) for circSMEK1 (red) in normal hepatocytes and HCC cells. Nuclei were stained with DAPI (blue). Scale bar, 10 µm. J) Serum circSMEK1 levels in Cohort 1: healthy controls (*n* = 10), MASH (*n* = 18), MASH‐HCC (*n* = 5), HCC (*n* = 20). K) Serum circSMEK1 levels in HCC patients with Vascular Invasion, (VI, *n* = 8) or without VI (Non‐VI, *n* = 12). L) Receiver operating characteristic (ROC) curve analysis of serum circSMEK1 for distinguishing HCC patients in Cohort 1 (AUC = 0.790, 95% CI [0.623 to 0.957]). M) Tissue circSMEK1 expression levels in Cohort 2: normal liver (*n* = 8), MASH‐HCC (*n* = 8), HCC (*n* = 20). N) circSMEK1 expression in paired peritumoral and HCC tissues from Cohort 3 (*n* = 55 pairs). O) ROC analysis of tissue circSMEK1 for discriminating HCC from peritumoral tissues in Cohort 3 (AUC = 0.742, 95% CI [0.646 to 0.839]). P) Kaplan–Meier survival analysis of HCC patients (Cohort 4) stratified by high vs low circSMEK1 expression (high vs low, total *n* = 206, *p =* 0.035). Q) Tissue circSMEK1 levels in HCC patients from Cohort 4 with (VI, *n* = 61) or without (Non‐VI, *n* = 145) vascular invasion. R) Representative Oil Red O staining images (left) and quantitative analysis of lipid content (right) in MASLD‐like and MASH‐like cell models following circSMEK1 knockdown (*n* = 3). Scale bar, 100 µm. S,T) Relative mRNA expression levels of *TNF‐α* and *TGF‐β* upon circSMEK1 knockdown in MASLD‐like and MASH‐like cell models (*n* = 3). U) circSMEK1 expression in normal hepatocyte cell lines (THLE‐2, THLE‐3), HCC cell lines (Huh‐7, SNU‐398, Hep3B), and hepatoblastoma cell line (HepG2) (*n* = 3). Data are presented as mean ± SD. *p*‐values were calculated using two‐tailed paired t‐test (C, N), two‐tailed unpaired t‐test (G, K, Q, R, S, T), one‐way ANOVA followed by Dunnett's multiple comparisons test (compared to the Normal group) (J, M), DeLong's test (L, O), Log‐rank test (*p*) ^*^ *p*< 0.05, ^**^ *p*< 0.01, ^***^ *p*< 0.001.

The progression from MASH to HCC reveals a direct pathological link with shared molecular dysregulation, offering potential biomarkers for early detection. To identify potential circRNAs dysregulated in both MASH‐related etiology and HCC, we analyzed transcriptomic datasets of circRNA from MASH (NASH) (GSE134146) and HCC (GSE164803, GSE97332) patients, identifying 8 promising candidate circRNAs: hsa_circRNA_ 102728, hsa_circRNA_101427, hsa_circRNA_100230, hsa_circRNA_ 104044, hsa_circRNA_104181, hsa_circRNA_101407, hsa_circRNA_104551, and hsa_circRNA_103554 (**Figure** **1**A,B). As the non‐invasive biomarker enhances the disease accessibility, thus using circAtlas, we found that only hsa_circRNA_101427 (circSMEK1) and hsa_circRNA_103554 (circTFRC) were detectable in blood (>2 counts per million, CPM) and liver tissue (>0.2 CPM) (Figure S1A, Supporting Information), suggesting their potential for non‐invasive detection. circSMEK1 was decreased in HCC, while circTFRC showed inconsistent expression in HCC tissues (*n* = 12) (Figure 1C), making it less reliable as a biomarker. Thus, we focused on circSMEK1 for further study.

circSMEK1 (717 nt) is derived from exons 3–4 of SMEK1 gene via back‐splicing (Figure 1D). Its back‐splicing junction (BSJ) was confirmed by sequencing (Figure 1E) and PCR assays with cDNA and genomic DNA (gDNA) primers in SNU‐398 cells (Figure 1F). Actinomycin D pulse‐chase assays revealed circSMEK1's greater stability compared to its linear mRNA counterpart (Figure 1G), and it was found to localize to both the cytoplasm and nucleus, as demonstrated by nuclear‐cytoplasmic fractionation assay (Figure 1H) and RNA fluorescence in situ hybridization (FISH) (Figure 1I).

Validation across four cohorts revealed: Cohort 1, serum circSMEK1 levels showed a declining trend in MASH (*n* = 18, *P* = 0.082) and were significantly lower in MASH‐HCC (*n* = 5, *P* = 0.0025) and HCC (*n* = 20, *P* = 0.0407), (Figure 1J). Low serum circSMEK1 correlated with higher vascular invasion (VI) in HCC patients (*n* = 20, *P* = 0.0441) (Figure 1K), and with an AUC of 0.790 (90% specificity, 75% sensitivity, 80% accuracy) in ROC analysis (Figure 1L). In cohort 2, circSMEK1 expression was reduced in MASH‐HCC (*n* = 8) and HCC (*n* = 20) tissues compared to controls (Figure 1M). Cohort 3 (55 paired HCC with para‐HCC) confirmed circSMEK1 downregulation in HCC (Figure 1N), with an AUC of 0.742 (80% specificity, 71% sensitivity) in ROC analysis, highlighting its diagnostic potential (Figure 1O). In cohort 4, high circSMEK1 expression correlated with better OS in 80 months (Figure 1P, FFPE‐HCC = 206, *P* = 0.035, HR = 1.62 [1.03–2.55]). The pathological characteristics of these patients are shown in **Table** **1**. Low circSMEK1 expression was associated with larger tumor Isize, VI, and Events (Table1 and Figure 1Q).

**Table 1:** Clinical features of cohort 4.

Characteristics	circSMEK1 high	circSMEK1 low	P value	statistic	method
**N**	103	103			
**Gender**			0.8639	0.0294	Chisq test
M	81 (39.3%)	82 (39.8%)			
F	22 (10.7%)	21 (10.2%)			
**Vascular invasion**			0.0221	5.2402	Chisq test
No	80 (38.8%)	65 (31.6%)			
Yes	23 (11.2%)	38 (18.4%)			
**Age, mean ± sd**	57.155 ± 9.4306	56.524 ± 10.166	0.6447	0.4619	T test
**Tumor size (cm)**			0.0366	4.3706	Chisq test
>5	45 (21.8%)	60 (29.1%)			
≤5	58 (28.2%)	43 (20.9%)			
**HBV**			0.8097	0.0580	Chisq test
Yes	94 (45.6%)	93 (45.1%)			
No	9 (4.4%)	10 (4.9%)			
**Bilirubin**[**≥ 1 mg dL^−1^**]			0.2638	1.2485	Chisq test
Yes	44 (21.4%)	52 (25.2%)			
No	59 (28.6%)	51 (24.8%)			
**Albumin[< 35 g L^−1^**]			0.5588	0.3418	Chisq test
No	86 (41.7%)	89 (43.2%)			
Yes	17 (8.3%)	14 (6.8%)			
**AFP [ng ml^−1^]**			0.3418	0.9035	Chisq test
>400	24 (11.7%)	30 (14.6%)			
≤400	79 (38.3%)	73 (35.4%)			
**Child‐Pugh score**			0.4774	0.5049	Yates' correction
A	103 (50%)	101 (49%)			
B	0 (0%)	2 (1%)			
**Events**			0.0443	4.0441	Chisq test
Death	32 (15.5%)	46 (22.3%)			
Recurrence	71 (34.5%)	57 (27.7%)			
**CNLC stage, n (%)**			0.7529	0.5677	Yates' correction
I	86 (42%)	85 (41.5%)			
II	13 (6.3%)	11 (5.4%)			
III	4 (2%)	6 (2.9%)			

Furthermore, we established in vitro models of MASLD^[^ ^14^ ^]^ and MASH.^[^ ^15^ ^]^ In the MASLD‐like model, knockdown (KD) of circSMEK1 did not significantly alter lipid accumulation (as assessed by Oil Red O staining, Figure 1R), or the expression of *TNF‐α* and *TGF‐β* (Figure 1S), as well as TG of cells and AST of supernatant (Figure S1B,C, Supporting Information), suggesting its limited role in the early steatotic stage. In contrast, in the MASH‐like model, circSMEK1 knockdown led to a significant increase in lipid accumulation (Figure 1R) and enhanced the expression of the pro‐inflammatory and pro‐fibrotic markers *TNF‐α* and *TGF‐β* (Figure 1T). These results indicate that circSMEK1 plays a specific and critical role in the progression from steatosis to inflammatory steatohepatitis (MASH), rather than in the initial lipid accumulation stage (MASLD).

Given circSMEK1's similar decreased expression in MASH‐HCC and non‐MASH‐HCC samples and lack of immortalized MASH‐like cell lines, we examined its expression in hepatocyte (THLE‐2, THLE‐3), HCC (SNU‐398, Huh‐7, Hep3B), liver cancer hepatoblastoma (HepG2) cells, finding it consistently downregulated in liver cancer cells (Figure 1U), a trend that aligns with the reduced signal observed by RNA FISH in HCC (Figure 1I).

In summary, circSMEK1 is downregulated in MASH‐HCC and HCC, correlates with HCC poor prognosis, and its serum level may have a non‐invasive biomarker potential for early HCC detection.

### 2.2 circSMEK1 Inhibits HCC Proliferation and Metastasis

**Figure 2:**
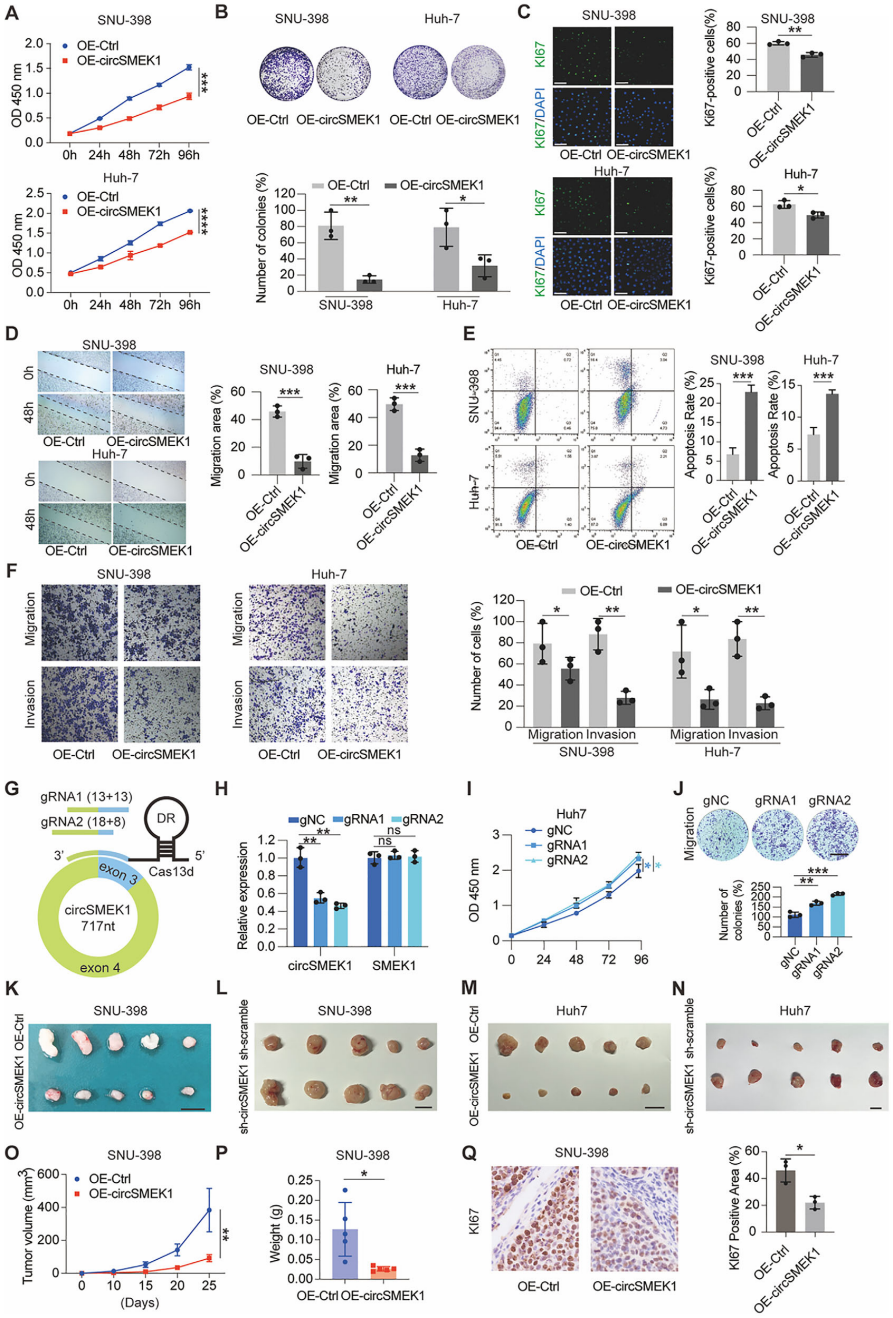
circSMEK1 inhibits HCC both in vitro and in vivo. A) CCK‐8 assay for proliferation in OE‐Ctrl and OE‐circSMEK1 HCC cells (*n* = 3). B) Colony formation assay for proliferation of OE‐Ctrl and OE‐circSMEK1 HCC cells (*n* = 3). C) Representative images and quantification of Ki‐67 staining for proliferation in OE‐Ctrl and OE‐circSMEK1 HCC cells (*n* = 3). D) Wound healing assay for migration in OE‐Ctrl and OE‐circSMEK1 HCC cells (*n* = 3). E) Flow cytometric analysis for apoptosis in OE‐Ctrl and OE‐circSMEK1 HCC cells (*n* = 3). F) Transwell migration and invasion assays of OE‐Ctrl and OE‐circSMEK1 HCC cells (*n* = 3). G) Schematic of the CRISPR‐RfxCas13d/BSJ‐gRNA system targeting the circSMEK1 back‐splicing junction (BSJ). H) Relative expression levels of circSMEK1 and linear *SMEK1* mRNA following BSJ‐targeted knockdown (*n* = 3). I) CCK‐8 assay assessing cell proliferation after circSMEK1 knockdown (*n* = 3). J) Transwell migration assay after circSMEK1 knockdown (*n* = 3). K–N) Representative images of xenograft tumors from nude mice implanted with control, OE‐circSMEK1, sh‐Ctrl, or sh‐circSMEK1 HCC cells (*n* = 5 mice per group). O) Tumor volume and measurements at endpoint in nude mice (*n* = 5 mice per group). P) Tumor weight quantification at endpoint (*n* = 5 mice per group). Q) Representative images and quantification of KI67‐positive areas in xenograft tumors (*n* = 3 fields per group). Data are presented as mean ± SD. *P*‐values were calculated by two‐tailed unpaired Student's t‐test (A, B, C, D, E, F, O, P, Q), One‐way ANOVA followed by Dunnett's multiple comparisons test (compared to the gNC group) (H, I, J) Significant results are presented as ^*^ *p* < 0.05, ^**^ *p* < 0.01, and ^***^ *p* < 0.001.

To investigate the effects of circSMEK1 on HCC, we overexpressed (OE) circSMEK1 in SNU‐398 and Huh‐7 cells. OE‐circSMEK1 significantly inhibited proliferation (**Figure** **2**A–C) and reduced migration (Figure 2D,F), enhanced the rate of apoptosis (Figure 2E), and reduced the invasion of HCC cells (Figure 2F). Using CRISPR‐RfxCas13d/BSJ‐gRNA to target the circSMEK1 BSJ locus (Figure 2G), ensured specifically reduced circSMEK1 expression in HCC cells without altering its cognate mRNA (Figure 2H). Knockdown (KD) circSMEK1 enhance proliferation (Figure 2I) and migration of HCC cells (Figure 2J).

OE‐circSMEK1 significantly inhibited tumorigenic phenotypes in nude mice (Figure 2K,M). KD‐circSMEK1 significantly promotes tumorigenic phenotypes in nude mice (Figure 2L,N). Consistently, the endpoint tumor volume, weight, and proliferation index based on KI67 staining were significantly reduced in OE‐circSMEK1 xenograft mice (Figure 2O–Q). Thus, OE‐circSMEK1 inhibited the proliferation, migration and invasion in HCC both in vitro and in vivo, and vice versa.

### 2.3 Nucleic‐circSMEK1 Binds hnRNPK Regulating IGF2/AKT Signaling in HCC

circRNAs often function as miRNA‐sponges in the cytoplasm. Ago2‐RIP experiments suggested that circSMEK1 may act as a miRNA‐sponge (Figure S2A, Supporting Information). Using CircInteractome and starBase, 4 potential target miRNAs were predicted, with hsa‐miR‐665 and hsa‐miR‐301a‐3p linked to OS of HCC patients in TCGA (Figure S2B,C, Supporting Information). Ago2‐RIP confirmed enrichment of both miRNAs (Figure S2D, Supporting Information). Luciferase assays showed that circSMEK1 specifically sponges hsa‐miR‐301a‐3p but not hsa‐miR‐665 (Figure S2E, Supporting Information). However, modulating hsa‐miR‐301a‐3p did not significantly affect proliferation of Hep3B and SNU‐398 cells (Figure S2F, Supporting Information), indicating that miRNA‐sponging is not circSMEK1's primary mechanism in HCC. Studies indicated that circRNAs can be translated into polypeptides in the cytoplasm.^[^ ^16^ ^]^ circSMEK1 was predicted to contain an internal open reading frame (iORF) (Figure S2G, Supporting Information) initiating with an “ATG” codon but lacking an in‐frame stop codon, theoretically encodes an endless protein with “239‐ amino‐acid” repeating. To validate this iORF, we constructed a circSMEK1‐3xFlag vector inserted immediately preceding the “ATG” to ensure the integrity of the iORF. A point‐mutation was also introduced within the iORF to disrupt the in‐frame reading (circSMEK1‐3XFlag‐Mut) (Figure S2H, Supporting Information). The results from transfected‐293T cells and immunoblotting (with anti‐Flag antibody) revealed no endless protein or ladder‐like bands (Figure S2I, Supporting Information). These findings suggest that circSMEK1's function is independent of canonical miRNA‐sponging or protein‐coding.

**Figure 3:**
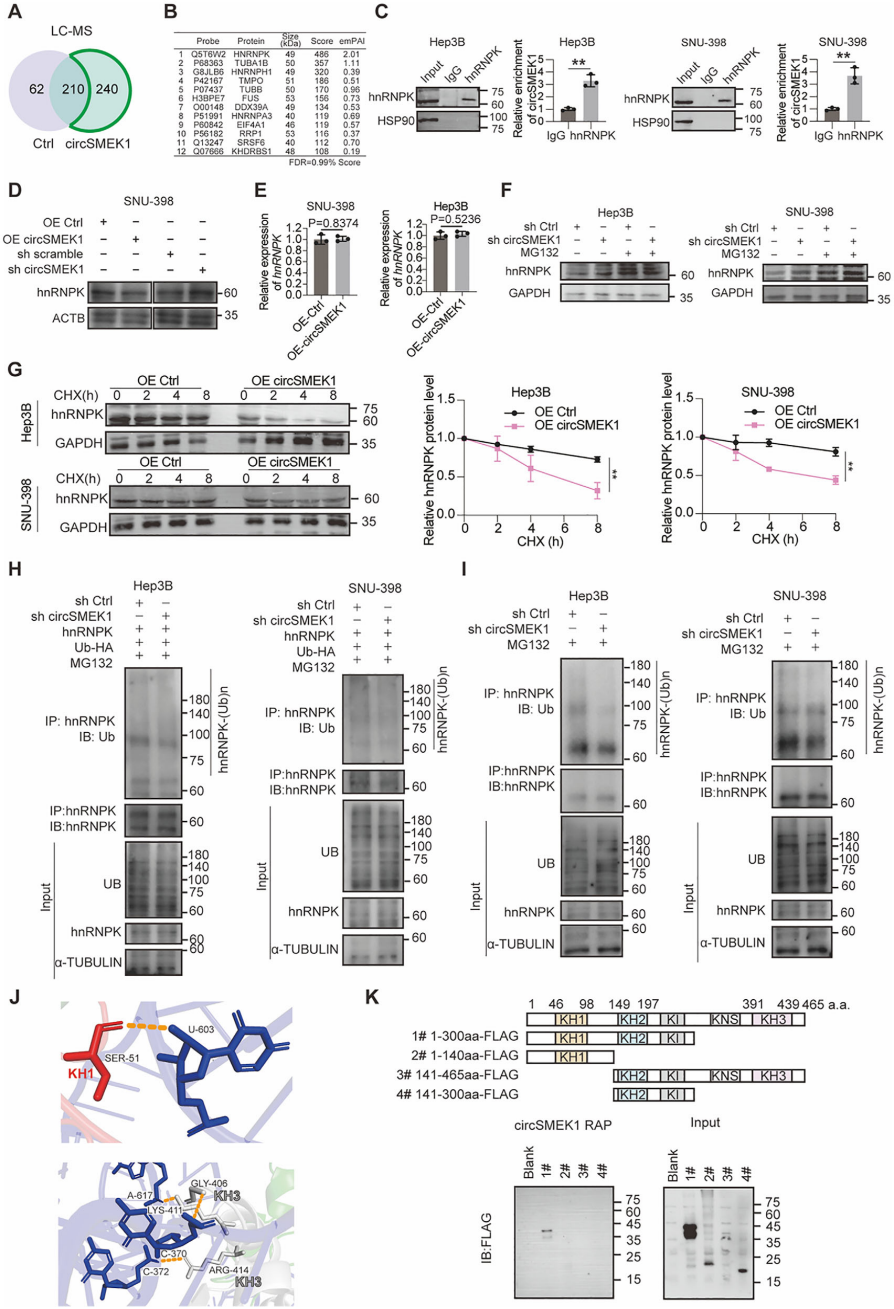
circSMEK1 Mediates hnRNPK Ubiquitination. A) Mass spectrometry analysis of proteins pull down by biotin‐labeled circSMEK1 probes. B) Top candidate proteins ranked by score. C) RNA immunoprecipitation assays test the binding of circSMEK1 to hnRNPK in HCC cells (*n* = 3). D) Protein level of hnRNPK with or without OE‐circSMEK1 or sh‐circSMEK1. E) Quantification of *hnRNPK* levels in OE‐Ctrl vs OE‐circSMEK1 HCC cells (*n* = 3). F) Protein level of hnRNPK in sh‐Ctrl and sh‐circSMEK1 cells treated with or without MG132 (*n* = 3). G) Cycloheximide (CHX) chase assay of hnRNPK stability in OE‐Ctrl and OE‐circSMEK1 HCC cells (*n* = 3). H,I) Immunoblot of hnRNPK ubiquitination in sh‐Ctrl and sh‐circSMEK1 cells with the treatment of MG132 to test exogenous H) and endogenous I) ubiquitination. J) AlphaFold‐3 prediction interaction sites with potential hydrogen bonds between the circSMEK1 sequence and KH1/KH3 domains of hnRNPK. K) Immunoblot for the FLAG‐tagged various domain‐truncated mutants of hnRNPK by RNA antisense purification by biotin‐labeled circSMEK1. Data are presented as mean ± SD. *p*‐values were calculated by two‐tailed unpaired *t*‐test (C, E, G). Significant results are presented as ^*^ *p* < 0.05, ^**^ *p* < 0.01, and ^***^ *p* < 0.001.

To identify alternative mechanisms of circSMEK1 in the nucleus, we performed CHIRP using a biotin‐labeled circSMEK1 probe. Compared to the controls, the specific enrichment of bands was ≈40–55 kDa (Figure S2J, Supporting Information). Mass spectrometry identified a series of circSMEK1‐binding proteins (**Figure** **3**A), among which hnRNPK ranks the first with the highest emPAI and Score (Figure 3B), and its mRNA level did not correlate with OS in HCC (P = 0.13, Figure S2K, Supporting Information). RIP‐assays confirmed the direct interaction between circSMEK1 and hnRNPK in Hep3B and SNU‐398 cells, respectively (Figure 3C).

OE‐circSMEK1 in HCC cells led to a decrease in hnRNPK level, whereas KD‐circSMEK1 resulted in increased hnRNPK level (Figure 3D). The consistent trend in hnRNPK levels was noted in both the nucleus and cytoplasm of cells (Figure S3A, Supporting Information), while *hnRNPK* mRNA remained unchanged (Figure 3E), indicating the predominant post‐transcriptional action of circSMEK1 on hnRNPK.

The ability of the proteasome inhibitor MG132 to further increase hnRNPK protein levels as it upregulated by sh‐circSMEK1 suggests that circSMEK1 promotes hnRNPK degradation via the proteasome pathway (Figure 3F). Moreover, Cycloheximide (CHX)‐chase experiments indicated that OE‐circSMEK1 shortened the half‐life of hnRNPK protein (Figure 3G). Further experiments demonstrated that KD‐circSMEK1 in cells inhibited the ubiquitination levels of exogenous hnRNPK (Figure 3H), and consistent with this, KD‐circSMEK1 also suppressed the ubiquitination of endogenous hnRNPK (Figure 3I). The KH domain, initially discovered in hnRNPK, is crucial for the binding RNA or single‐stranded DNA.^[^ ^17^ ^]^ Using AlphaFold‐3, we predicted that the key binding sites of hnRNPK with circSMEK1 sequence are in the KH1 and KH3 domains (Figure 3J). Subsequently, RAP (RNA‐Affinity Purification) assay indicated that only KH1 domain of hnRNPK possesses the capacity to bind circSMEK1 (Figure 3K).

**Figure 4:**
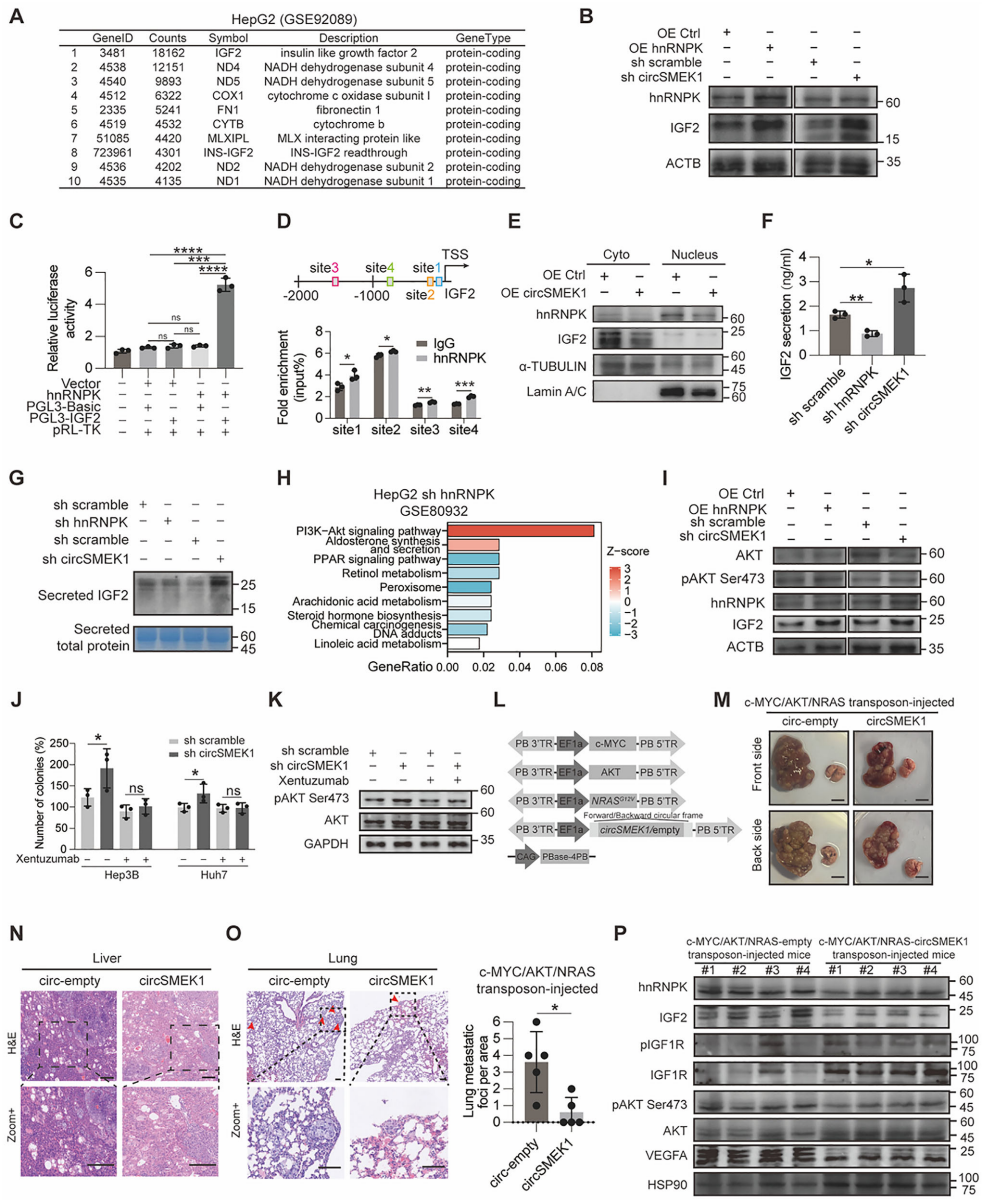
Nucleic circSMEK1 interacts with hnRNPK to regulate IGF2/AKT demonstrates therapeutic potential for HCC. A) IGF2 ranked as the top protein‐coding gene in hnRNPK eCLIP‐seq analysis. B) Western blot analysis of hnRNPK and IGF2 protein levels in HCC cells with or without hnRNPK overexpression or circSMEK1 knockdown. C) Dual‐luciferase reporter assay assessing hnRNPK activity on the 5′ UTR of IGF2 (*n* = 3). D) Chromatin immunoprecipitation (ChIP) qPCR showing hnRNPK enrichment at the −2000 bp region upstream of the IGF2 transcription start site (*n* = 3). E) Western blot of hnRNPK and IGF2 in nuclear and cytoplasmic fractions of HCC cells with or without circSMEK1 overexpression. F) ELISA measuring IGF2 levels in cell culture supernatant from cells with or without sh hnRNPK or sh circSMEK1 (*n* = 3). G) Western blot analysis of secreted IGF2 by supernatant protein extraction from cells with or without sh hnRNPK or sh circSMEK1 (*n* = 3). H) KEGG pathway analysis of differentially expressed genes from HepG2 cells with hnRNPK knockdown. I) Western blot of hnRNPK, IGF2, and phosphorylated AKT (p‐AKT) in HCC cells with or without hnRNPK overexpression or circSMEK1 knockdown. J) Colony formation assay of HCC cells treated with sh‐circSMEK1 and/or Xentuzumab (0.5 µM) (*n* = 3). K) Western blot analysis of p‐AKT levels in HCC cells treated with sh‐circSMEK1 and/or Xentuzumab (0.5 µM) (*n* = 3). L) Schematic of the c‐MYC/AKT/NRAS PiggyBac transposon system for hydrodynamic tail vein injection in mice with or without circSMEK1 overexpression. M) Representative images of livers and lungs from transposon‐mediated mouse models (*n* = 5 mice per group). N) Representative H&E staining of liver sections from transposon‐mediated mice with or without circSMEK1 overexpression (*n* = 5 mice per group). O) Representative H&E staining (left) and quantification (right) of lung metastatic foci in transposon‐mediated mice with or without circSMEK1 overexpression (n = 5 mice per group). P) Western blot analysis of hnRNPK, IGF2, p‐AKT, p‐IGF1R, and VEGFA protein levels in liver tissues from the transposon‐mediated mouse model (n = 4 randomly chosen from 5 biological replicates). Data are presented as mean ± SD. *p*‐values were calculated by two‐tailed unpaired (D, J, O) or One‐way ANOVA followed by Dunnett's multiple comparisons test (C, F). Significant results are presented as ^*^ *p* < 0.05, ^**^ *p* < 0.01, and ^***^ *p* < 0.001.

eCLIP‐seq is a valuable tool for probing functions of the RNA‐binding proteins (RBPs). Re‐analyzing eCLIP‐seq data for hnRNPK from HepG2 cells (GSE92089), we identified insulin‐like growth factor 2 (IGF2) with the highest read‐counts among protein‐coding genes, which interacts with hnRNPK (**Figure** **4**A).

Subsequently, both KD‐circSMEK1 and OE‐hnRNPK led to increased IGF2 protein levels in HCC cells (Figure 4B). Furthermore, ChIP‐seq analysis for hnRNPK in HepG2 (GSE174872) cells revealed an enrichment peak at 2000 bp upstream of the transcription start site (TSS) of *IGF2*, suggesting its possible transcriptional‐regulating role for *IGF2* (Figure S3B, Supporting Information). Dual‐luciferase assays further demonstrated that hnRNPK exhibits higher relative fluorescence activity with the 5′ UTR of *IGF2* (Figure 4C). ChIP‐qPCR confirmed that hnRNPK enriched at 2000 bp upstream of the TSS of *IGF2* in HCC cells (Figure 4D). These findings suggest that hnRNPK serves as a transcription factor for *IGF2*.

Nuclear‐cytoplasmic fractionation experiments of OE‐circSMEK1 HCC cells revealed that hnRNPK and IGF2 proteins were downregulated in both the nucleus and cytoplasm, with hnRNPK primarily distributing in the nucleus and IGF2 predominantly presenting in the cytoplasm (Figure 4E). To investigate the impact of hnRNPK and circSMEK1 on secreted IGF2 in vitro, we measured the level of IGF2 in the culture supernatant. KD‐hnRNPK reduced IGF2 level in the culture supernatant, while OE‐circSMEK1 suppress IGF2 level (Figure 4F,G) with a time‐dependent accumulation within 48 h (Figure S3C, Supporting Information). Furthermore, by re‐analysis of the transcriptome data on KD‐hnRNPK‐HepG2 cells (GSE80932), we found that differentially expressed genes were significantly enriched in the PI3K‐AKT pathway (Figure 4H). Additional experiments demonstrated that KD‐circSMEK1 or OE‐hnRNPK could both elevate IGF2 protein levels and increase the phosphorylated‐AKT in HCC (Figure 4I).

In summary, circSMEK1 primarily exerts its action by directly interacting with nucleic hnRNPK, which regulates the transcription of IGF2, thereby affects AKT pathway in HCC.

### 2.4 circSMEK1 has the Potential as a Therapeutic Strategy for HCC

An alternative therapeutic strategy targeting the inhibition of the IGF pathway, such as Xentuzumab (BI 836 845, a recombinant‐humanized monoclonal antibody for IGF),^[^ ^18^ ^]^ has emerged as the promising treatment for solid tumor in phase I and II. In HCC cells, sh‐circSMEK1 alone increased HCC clone numbers, this increased effect could be counteracted by combination of sh‐circSMEK1 with Xentuzumab (Figure 4J). Furthermore, the impact of IGF2, and Xentuzumab on HCC and their effects on phosphorylated‐AKT were examined (Figure 4K).

Given that activated‐Akt signaling is prerequisite for vascular endothelial growth factor (VEGF) production of HCC cells and angiogenesis for HCC,^[^ ^19^ ^]^ and circSMEK1 expression was negatively associated with VI in Cohort 4 (Figure 1O and Table 1).

As 93% sequence similarity in the exon 4–5 of *SMEK1* between humans and mice, suggests a possible similar role of circSMEK1 across species (Figure S3D, Supporting Information). Using our previously established PiggyBac‐system,^[^ ^20^ ^]^ we investigated the effects of OE‐circSMEK1 on HCC in mice (Figure 4L). OE‐circSMEK1 significantly inhibits HCC in liver (Figure 4M,N) and lung metastasis (Figure 4O) in PiggyBac‐mediated mice. Moreover, the lower levels of hnRNPK and IGF2, as well as the reduced levels of phosphorylated‐AKT, phosphorylated‐IGF1R and VEGFA were observed in the liver of OE‐circSMEK1 mice (Figure 4P).

Taken together, these results indicated that restoring circSMEK1 has strong potential as a therapeutic strategy for HCC.

### 2.5 SF3B4 Drives Downregulation of circSMEK1 in HCC

**Figure 5:**
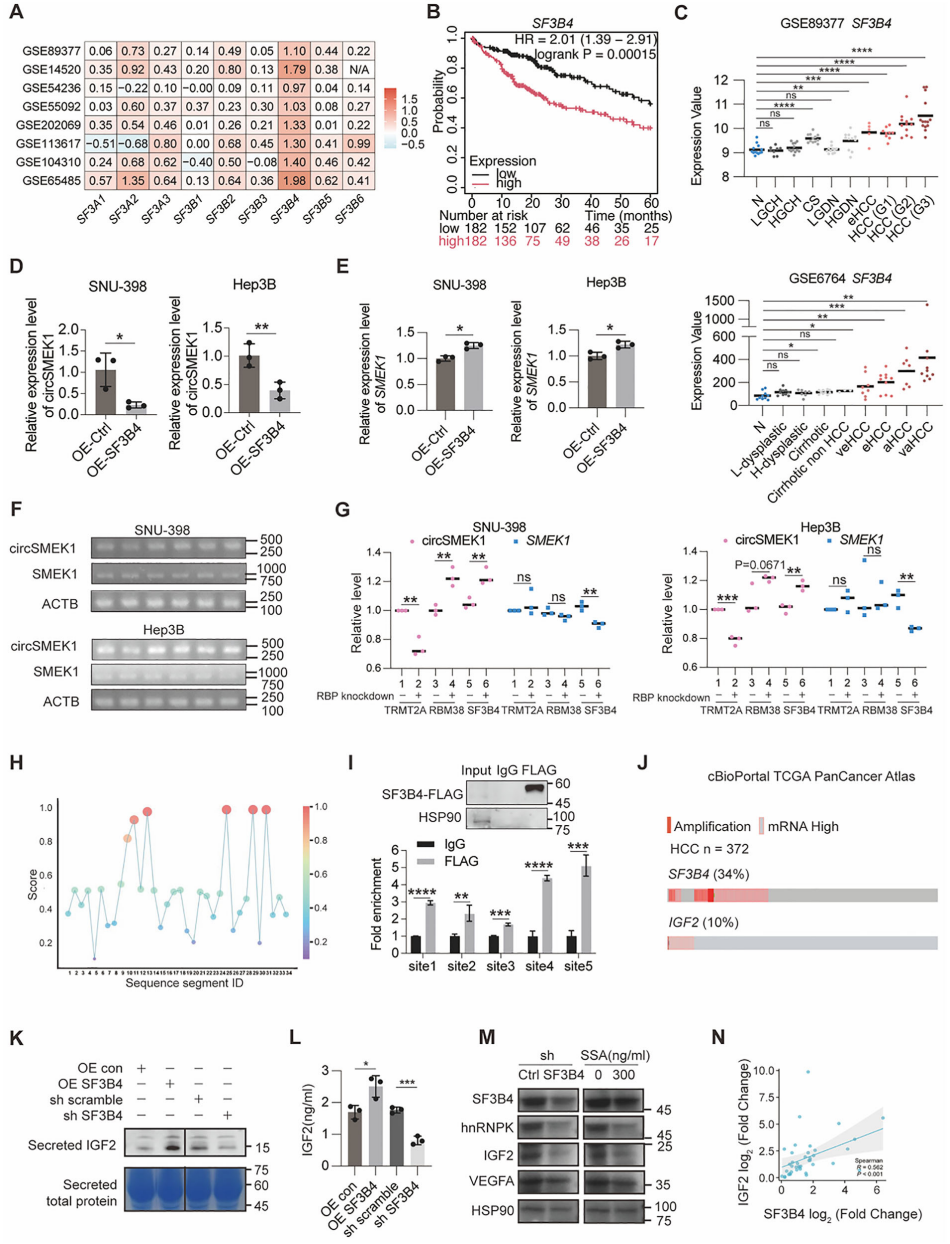
SF3B4 regulates the biogenesis of circSMEK1 in HCC. A) Expression of 9 members of the SF3A and SF3B complex components across 8 large HCC cohorts from GEO datasets. B) Kaplan‐Meier survival analysis of HCC patients based on SF3B4 expression from the TCGA database. C) Re‐analysis of stratified samples from chronic liver disease and different stages of HCC in GSE89377 and GSE6764 datasets. D) Relative expression of circSMEK1 in HCC cells with or without SF3B4 overexpression (*n* = 3). E) Relative expression of linear SMEK1 mRNA in HCC cells with or without SF3B4 overexpression (*n* = 3). F,G) Semi‐quantitative RT‐PCR analyzed by agarose gel electrophoresis showing relative levels of circSMEK1 and SMEK1 in HCC cells following RBP knockdown of TMRT2A, RBM38, or SF3B4 (*n* = 3). H) Predicted SF3B4 binding sites within the 1000 nt upstream and downstream flanking regions of circSMEK1. I) RNA immunoprecipitation (RIP) assays testing SF3B4 binding affinity at 5 predicted sites within the flanking regions of circSMEK1 (*n* = 3). J) Cooperative relationship analysis of genetic alterations in SF3B4 and IGF2 in HCC patients from TCGA PanCancer Atlas (*n* = 372). K) Western blot analysis of secreted IGF2 by supernatant protein extraction from cells with or without OE SF3B4 or sh SF3B4 (*n* = 3). L) ELISA measuring IGF2 levels in cell culture supernatant from cells with or without OE SF3B4 or sh SF3B4 (*n* = 3). M) Western blot analysis of SF3B4, hnRNPK, IGF2, and VEGFA in cells with or without sh SF3B4 or treatment with Spliceostatin A (300 ng mL^−1^) (*n* = 3). N) Correlation analysis between IGF2 and SF3B4 expression in Cohort 2 individuals with the lowest 20% circSMEK1 expression levels (R = 0.562, *n* = 40). Data are presented as mean ± SD. *P*‐values were calculated by unpaired two‐tailed t‐test (D, E, G, I, L), log‐rank test (B), or one‐way ANOVA followed by Dunnett's multiple comparisons test (vs the N group) (C). Correlation was analyzed by Spearman correlation analysis (N). Significant results are presented as ^*^ *p* < 0.05, ^**^ *p* < 0.01, and ^***^ *p* < 0.001.

To further elucidate the mechanism underlying the downregulation of circSMEK1 in HCC, we first searched TCGA for the expression of its cognate mRNA and its correlation with the prognosis of HCC patients. Surprisingly, in paired adjacent and cancerous tissues of HCC, *SMEK1* mRNA was significantly upregulated in the cancerous tissues (Figure S4A, Supporting Information), and this higher expression of *SMEK1* was associated with the shorter OS of HCC patients (Figure S4B, Supporting Information), which is opposite to the trend observed for circSMEK1 in this study (Figure 1P). Although the levels of circRNAs and their cognate linear mRNAs are generally uncorrelated, components of the U2 snRNP—including the SF3A and SF3B complexes (core spliceosomal factors)—have been reported to balance their production through a “compensatory see‐saw” mechanism.^[^ ^21^ ^]^ We therefore propose that such a mechanism may contribute to circSMEK1 biogenesis. Analysis of nine SF3A and SF3B subunits across eight large HCC cohorts from GEO revealed significant upregulation of SF3B4, suggesting a specific role in HCC (**Figure** **5**A). Higher SF3B4 expression was associated with poorer OS in the TCGA‐LIHC cohort (Figure 5B). Re‐analysis of stratified samples of diverse liver diseases from GSE89377 and GSE6764 further supported a potential role for SF3B4 as a key driver in HCC progression (Figure 5C).^[^ ^22^ ^]^ In support of our hypothesis, circAtlas 2.0 (January 2024 snapshot, Figure S4C, Supporting Information) predicted SF3B4 binding within the flanking region of circSMEK1.

To investigate whether SF3B4 inhibits circSMEK1 biogenesis, we OE‐SF3B4 in HCC cells, observing significant downregulation of circSMEK1 (Figure 5D) and upregulation of *SMEK1* mRNA (Figure 5E). Given that TRMT2A and RBM38 were identified in a prior genome‐wide shRNA screen as modifiers of circRNA abundance without affecting their linear counterparts,^[^ ^23^ ^]^ we next performed semi‐quantitative RT‐PCR in HCC cells following knockdown of selected RNA‐binding proteins (RBPs)—SF3B4, TRMT2A, and RBM38 (Figure 5F) (TRMT2A and RBM38 were included as controls). Notably, SF3B4 knockdown increased circSMEK1 levels but decreased linear SMEK1 mRNA (Figure 5F,G), further supporting the hypothesis that SF3B4 specifically suppresses circSMEK1 biogenesis while promoting canonical splicing of its host gene.

To further explore the inhibitory mechanism of SF3B4 on circSMEK1, we predicted SF3B4 binding sites within a 1000‐nt upstream and downstream of circSMEK1, and the top 5 binding sites were further examined (Figure 5H Figure S4D,E, Supporting Information). RIP‐assay confirmed varying binding affinity at 5 sites on flanking of circSMEK1 (Figure 5I).

Using the cBioPortal database, we analyzed genetic alterations in SF3B4 (amplified in 34% of HCC) and IGF2 (amplified in 10% of HCC) from TCGA PanCancer Atlas (*n* = 372). The results showed that co‐amplification occurred in ≈5% of cases, suggesting a cooperative relationship (Figure 5J). OE‐ or KD‐SF3B4 in HCC cells increased or decreased secreted IGF2 levels, respectively (Figure 5K,L).

Spliceostatin A (SSA) / FR901464, an SF3B complex inhibitor, was used to further illustrate the SF3B4‐circSMEK1‐hnRNPK‐IGF2‐AKT axis. Protein analysis confirmed that KD‐SF3B4 or SSA treatment altered circSMEK1 downstream hnRNPK, IGF2, and VEGFA levels (Figure 5M).

We further investigated the correlation between SF3B4 and IGF2 level in HCC tissues from in Cohort 2 individuals with the lowest 20% circSMEK1 expression levels (R = 0.562, *p*<0.001, *n* = 40) (Figure 5N) as well as TCGA (R = 0.108, P = 0.036, Figure S4F, Supporting Information).

In summary, SF3B4 regulates circSMEK1 biogenesis, influencing the SF3B4‐circSMEK1‐hnRNPK‐IGF2‐AKT axis in HCC.

### 2.6 Decreased circSMEK1 Lifts IGF2 Secretion from HCC Cells, Activate IGF2/AKT in CAFs Through Paracrine

**Figure 6:**
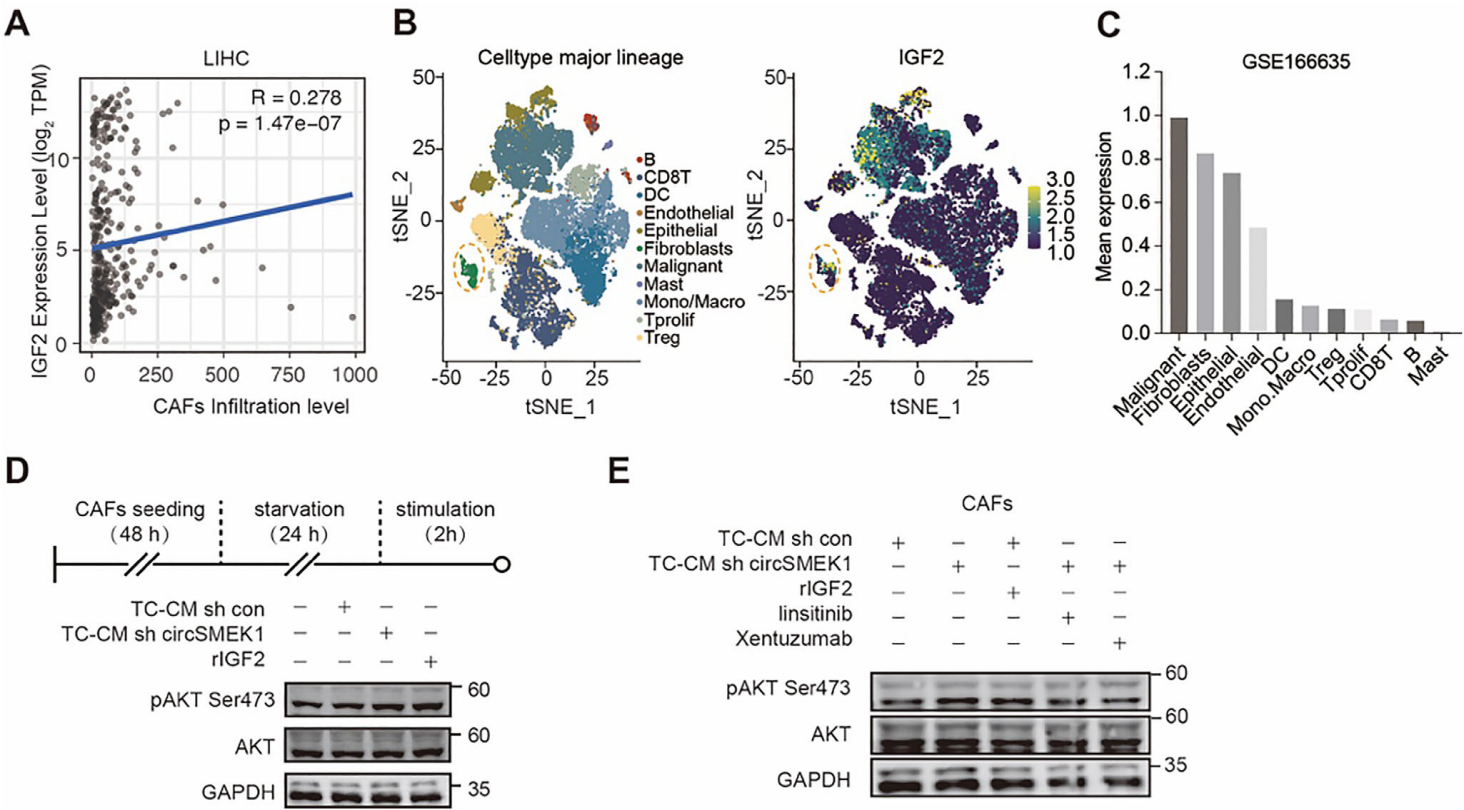
Low levels of circSMEK1 enhance IGF2 secretion of HCC tumor cells activating cancer‐associated fibroblasts (CAFs). A) Analysis from TIMER database showing a positive correlation between IGF2 and the infiltration of CAFs in TME of liver hepatocellular carcinoma (LIHC). B,C) Single‐cell RNA sequencing of IGF2 expression level among different groups of cells in TME cell clusters of HCC (GSE166635). D) Western blot analysis of phosphorylated‐AKT in CAFs, with tumor cell‐conditional medium (TC‐CM) from HCC cells with or without sh circSMEK1 or stimulated with exogenous recombinant‐IGF2 E. Western blot analysis of phosphorylated‐AKT in CAFs, with tumor cell‐conditional medium (TC‐CM) from HCC cells with or without sh circSMEK1 or stimulated with exogenous recombinant‐IGF2, IGF1R TKI inhibitor Linsitinib (1 µM), or IGF1/2 mAb Xentuzumab (0.5 µM). *p*‐values were calculated by log‐rank test (A).

As IGF2 is a secretory protein, which promotes tumor invasiveness and stemness in TME.^[^ ^24^ ^]^ To further investigate the effects of IGF2 on TME, through TIMER database, we found that *IGF2* expression was positively correlated with infiltrated cancer‐associated fibroblasts (CAFs) than rest cell types of TME in HCC (**Figure** **6**A). Re‐analysis of *IGF2* expression from scRNA‐seq in TME cell clusters of HCC (GSE166635) shown that *IGF2* was most abundant in HCC cells, with also notably high espression in CAFs (Figure 6B,C), implying a fundamental role of IGF2 in HCC malignancy. Since IGF2 is mainly secreted by HCC cells and can be detected in culture supernatant (Figures 4J and 5K), we then checked the paracrine effect of IGF2 on CAFs isolated from PiggyBac‐HCC mice (Figure S5A, Supporting Information). Increased levels of p‐AKT in starved CAFs were induced by treatment with tumor cell‐conditioned medium (TC‐CM) from KD‐circSMEK1 HCC cells—which contains elevated IGF2 due to enhanced secretion—or by direct stimulation with exogenous recombinant IGF2. These results suggest that HCC‐derived IGF2 mediates activation of the IGF2/AKT pathway in CAFs (Figure 6D). Moreover, this enhancement was further exacerbated by treatment with exogenous recombinant IGF2, but could be effectively reversed by either the IGF1/2 neutralizing antibody Xentuzumab or the IGF1R tyrosine kinase inhibitor Linsitinib (Figure 6E). These findings further support the conclusion that circSMEK1 knockdown in HCC cells can affect and promote IGF2/IGF1R‐mediated AKT activation in CAFs. Therefore, HCC‐derived IGF2 from KD‐circSMEK1 activates the IGF2/AKT axis in CAFs via paracrine secretion, resulting in immunosuppressive microenvironment.

## 3 Discussion

Given that MASH‐HCC patients exhibit reduced survival with ICIs, over 38% MASH cases progress directly to HCC underscores a direct pathological‐link between MASH and HCC.^[^ ^11^ ^]^ These highlight the urgent need for novel biomarker‐based stratification for HCC. Our study identifies circSMEK1, downregulated in MASH and HCC, may as a tumor suppressor whose loss correlates with tumor size, VI, and poor OS, suggesting its potential as a perspective serum biomarker and therapeutic target for HCC. Mechanistically, circSMEK1 suppresses tumorigenesis by directly binding hnRNPK to inhibit IGF2/AKT signaling and indirectly remodeling TME.

### 3.1 circSMEK1 as a Diagnostic and Prognostic Biomarker

The downregulation of circSMEK1 in tumor and serum of HCC patients underscores its potential as a non‐invasive biomarker for detection. Its serum diagnostic potential is supported by an AUC of 0.790 in ROC analysis, with high specificity and sensitivity. Furthermore, low circSMEK1 correlates with worse OS of HCC patients. These findings align with the growing interest in circRNAs as stable, tissue‐specific biomarkers for cancer diagnosis and prognosis. Its detectability in blood further enhances its clinical utility for HCC screening and monitoring. Although serological markers and imaging techniques (such as multi‐parametric MRI and ultrasound elastography) can assist in detecting liver fibrosis and MASH, their performance varies significantly, liver‐biopsy remains the current “gold standard” for diagnosing MASH. However, due to the invasive nature of biopsy, making it difficult to widely apply in clinical practice. Currently, there is no outstanding non‐invasive indicator capable of distinguishing MASH from steatosis. We identify circSMEK1 as significantly downregulated in the serum of MASH and HCC patients, highlighting its biomarker potential for MASH‐HCC transition. We emphasize that such confirmation in larger, multi‐center prospective cohorts is an essential requirement for any HCC diagnostic biomarker, and it holds particular importance for early HCC detection. This is because early‐stage lesions often present with non‐specific clinical and imaging features, making it imperative that any proposed biomarker demonstrates robust performance across diverse populations in distinguishing early HCC from benign liver conditions (such as “Cirrhosis no HCC” controls, similar to the stratified analysis we performed for SF3B4 in the Figure 5C) through rigorous multi‐center validation before any clinical application need to be considered in future. Also, the limited number of pure MASH and MASH‐HCC cases in this study reduces statistical power, impacting its reliability and validity. To validate circSMEK1 as a biomarker and therapeutical target for MASH and HCC, large‐scale, prospective, multicenter, double‐blind clinical trials are warranted.

### 3.2 Mechanistic Actions and Therapeutic Implications of circSMEK1

In our study, we predicted a junction‐spanning ORF and a potential IRES within circSMEK1 but detected no novel peptides. This could be due to insufficient IRES activity, low translation efficiency influenced by RBPs, or rapid peptide degradation. Similarly, circSMEK1 was identified as a potential miRNA‐sponge, with Ago2‐RIP and luciferase assays confirming interaction with hsa‐miR‐301a‐3p, whereas modulating hsa‐miR‐301a‐3p failed to affect HCC proliferation or invasion. These findings suggest that circSMEK1's action in cytosol is limited.

Experiments revealed that circSMEK1 directly interacts with the KH domain of hnRNPK, promoting its ubiquitin‐mediated degradation. eCLIP‐seq analysis identified this circSMEK1‐hnRNPK interaction regulating *IGF2* expression and subsequently suppressing the PI3K/AKT pathway, thereby inhibiting HCC progression. These findings highlight circSMEK1's nucleic role and offer a potential therapeutic target. Beyond its cell‐autonomous effects, circSMEK1 influences TME by regulating IGF2 secretion. Reduced circSMEK1 increase autocrine IGF2, which in turn activates PI3K/AKT signaling in CAFs, promoting an immunosuppressive microenvironment. This paracrine effect of IGF2 on CAFs underscores the broader impact of circSMEK1 dysregulation on tumor progression and highlights a potential therapeutic target within TME.

The therapeutic potential of circSMEK1 is further supported by its capacity to inhibit HCC growth and metastasis in PiggyBac‐mediated mice. OE‐circSMEK1 significantly reduces tumor volume and weight, suppresses HCC cell proliferation and lung metastasis in mice. Additionally, the combination of OE‐circSMEK1 with IGF inhibitor, such as Xentuzumab, demonstrates synergistic anti‐tumor effects, suggesting a promising strategy for HCC treatment.

### 3.3 SF3B4 Regulates circSMEK1 Biogenesis

Consistent with the previous study,^[^ ^22^ ^]^ we found that SF3B4 negatively regulates circSMEK1 biogenesis in HCC. The “compensatory see‐saw effect”^[^ ^21^ ^]^ between circSMEK1 and its cognate mRNA, highlights the complex regulatory networks governing circRNA expression. Targeting SF3B4 could offer new avenue for restoring circSMEK1 and suppressing HCC progression.

In conclusion, our study has shed light on the roles of circSMEK1 in the context of MASH/HCC, linked to disease progression and survival. circSMEK1 interacts with hnRNPK to modulate the IGF2‐AKT pathway suppressing HCC development.

## 4 Experimental Section

### Ethical Approval and Study

This study integrates retrospective analyses of clinical cohorts, in vitro and in vivo models. Human serum and tissue were collected from patients with MASH, HCC, and healthy‐controls across four independent cohorts (Cohort 1–4). Human samples were collected same way as our previous study,^[^ ^25^ ^]^ which were obtained with informed consent under protocols approved by the Ethical Committee at Shandong Provincial Hospital (SWYX‐No. 2022–293) and Yuhuangding Hospital (No.2024–511).

Animal studies (No. AW02604202‐3‐1, AW52213202‐3‐3, AW22505202‐3‐01) were approved by the Ethics Committee of the China Agricultural University.

### Clinical Cohorts and Sample Preparation

Cohort 1: Human serum samples (MASH = 18, MASH‐HCC = 5, HCC = 20 and healthy‐control = 10). Cohort 2: Fresh liver samples (Normal‐control = 8, MASH‐HCC = 8, HCC = 20). Cohort 3: HCC and adjacent non‐tumor tissues (*n* = 55 paired). Cohort 4: Formalin‐fixed paraffin‐embedded (FFPE) tissues from patients with follow‐up data (HCC = 206).

For MASH‐HCC samples from patients without the history of viral‐hepatitis, autoimmune disease, cholestatic disease, alcohol‐related liver disease, or drug‐induced liver injury were collected. Liver normal‐controls were obtained from hepatic hemangioma surgeries, FFPE‐HCC samples were collected between 2017–2018.

Serum was processed by centrifugation (3000 × g, 10 min) and stored at −80 °C. Tissues were snap‐frozen in liquid nitrogen or fixed in 4% paraformaldehyde.

### HCC‐Xenograft Mouse Model

Five‐week‐old male BALB/c nude (nu/nu) mice were purchased from SPF Biotechnology (Beijing, China) and kept in SPF condition. ≈1*10^6^ mL^−1^ cells with Matrigel GelNest (211 262, NEST, Jiangsu, China) and DMEM (1:1) were injected subcutaneously into mice after acclimatization. Mice were sacrificed before tumors exceeding 2 cm. Tumors were weighed and fixed in 4% paraformaldehyde.

### Genetical‐Engineered Mouse Model

HCC mosaic‐mouse was generated using our previous PiggyBac‐system.^[^ ^18^, ^20^ ^]^ Six‐week‐old male C57BL/6 received tail‐vein injection with plasmid: Transposons‐encoding for c‐MYC (0.3 µg), AKT (0.1 µg), NRAS^G12V^ (0.1 µg), circSMEK1 (5 µg) or respective empty transposon, PBase (0.1 µg) to each mouse, respectively. Linear circSMEK1 with forward/backward circular‐frame was generated from pLO5‐ciR plasmid. Plasmids were diluted in 0.9% NaCl and injected at a volume of 10% of body‐weight into tail‐vein.

### Cell Lines and Culture

HCC cell lines (SNU‐398, Huh‐7, Hep3B), liver cancer hepatoblastoma (HepG2), immortalized normal hepatocytes (THLE‐2, THLE‐3) were obtained from ATCC or the Chinese Academy of Sciences. Cells were cultured in DMEM supplemented with 10% FBS and 1% penicillin/streptomycin at 37 °C with 5% CO_2_. Mycoplasma contamination was routinely tested by MycoAlert Kit (Lonza, Cologne, Germany).

### Statistical Analysis

Statistical analysis was performed using GraphPad Prism software (version 10.1). Data are presented as mean ± SD. The specific statistical tests applied in each experiment are detailed in the corresponding figure legends. These included two‐tailed paired t‐test, two‐tailed unpaired t‐test, one‐way ANOVA followed by Dunnett's multiple comparisons test, DeLong's test, Log‐rank test, and Spearman correlation analysis. Statistical significance was defined as ^*^ *p* < 0.05, ^**^ *p* < 0.01, and ^***^ *p* < 0.001. The exact sample size (n) for each experiment, representing biological replicates, is provided in the figure legends.

### Declaration of Generative AI and AI‐Assisted Technologies in the Writing Process

During the preparation of this work the author used DeepSeek in order to editing the language. After using this tool/service, the author reviewed and edited the content as needed and take full responsibility for the content of the publication.

## Conflict of Interest

The authors declare no conflict of interest.

## Author Contributions

P.G., X.J., S.W., X.L., and Y.L. are co‐first authors and contributed equally to this work. P.G., Y.L., J.L., and X.L. contributed conceptualization. P.G., X.J., S.W., and Y.L. managed data curation. P.G., Y.L., L.L., and Z.W. performed formal analysis. X.L. handled funding acquisition. P.G., X.J., S.W., and Y.L. performed investigation. P.G., Y.L., S.W., N.R., F.T., J.L., X.L. managed methodology. P.G., J.L., X.L. managed project administration. X.L., J.G., W.S., F.L., X.D., S.T., W.S. handled resources. P.G., L.L., Z. W. handled software. S.T., W.S., J.L., X.L. handled supervision. P.G., X.J., S.W., Y.L. performed validation. P.G., Y.L., L.L., Z.W. contributed Visualization. P.G., X.L. contributed writing – original draft. P.G., S.T., J.L., X.L. handled writing – review & editing.

## Supporting information



Supporting Information

## Data Availability

The authors declare that the data supporting the results of this study are available within the paper and Supplementary files. All data supporting this work are available from the corresponding author on reasonable request.
