# Supplementary material for: CircSMEK1 Suppresses HCC via the hnRNPK‐IGF2‐AKT Axis: A Diagnostic Biomarker and Therapeutic Target
Source: Adv Sci (Weinh). 2025 Oct 17;12(48):e05267. doi: 10.1002/advs.202505267 (PMC12752665; doi:10.1002/advs.202505267)
Supplement: Supplementary file 1 — Supporting Information [file ADVS-12-e05267-s001.docx]

**CircSMEK1 Suppresses HCC via the hnRNPK-IGF2-AKT Axis: A Diagnostic Biomarker and Therapeutic Target**

Peilan Guo^#^, Xiaomeng Jia^#^, Shenghong Wang^#^, Xinyu Li^#^, Yajing Liu^#^, Lisen Lin, Zhengkun Wang, Fujun Liu, Slawomir Wolczynski, Nafis Rahman, Jie Gao，Xuguang Du, Suk-Ying Tsang*, Jiali Liu*, Wei Song*, Xiangdong Li*

**Supplementary material**

**Page 2-6 Supplementary Figure**

**Page 7-13 Additional methods**

**
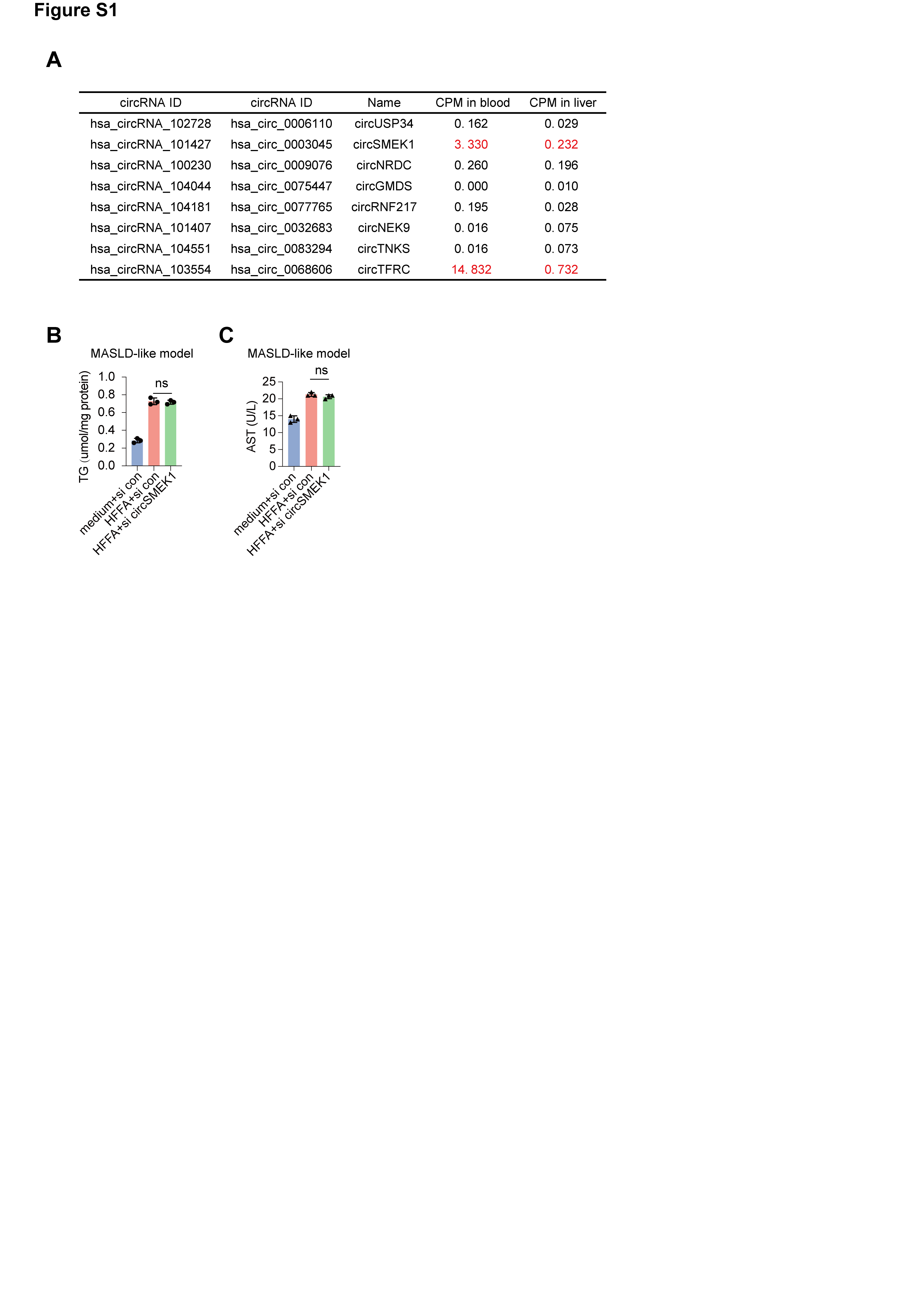
**

**Figure S1 circRNA concentration in blood and liver**

**A.** circRNA concentration in blood and liver for circAtlas, CPM, Count per million. **B.** TG content of cells of the MASLD-like model **C.** AST content of the supernatant of the MASLD-like model. Data are presented as mean ± SD. P-values were calculated by unpaired two-tailed t-test. Significant results are presented as *P < 0.05, **P < 0.01, and ***P < 0.001.


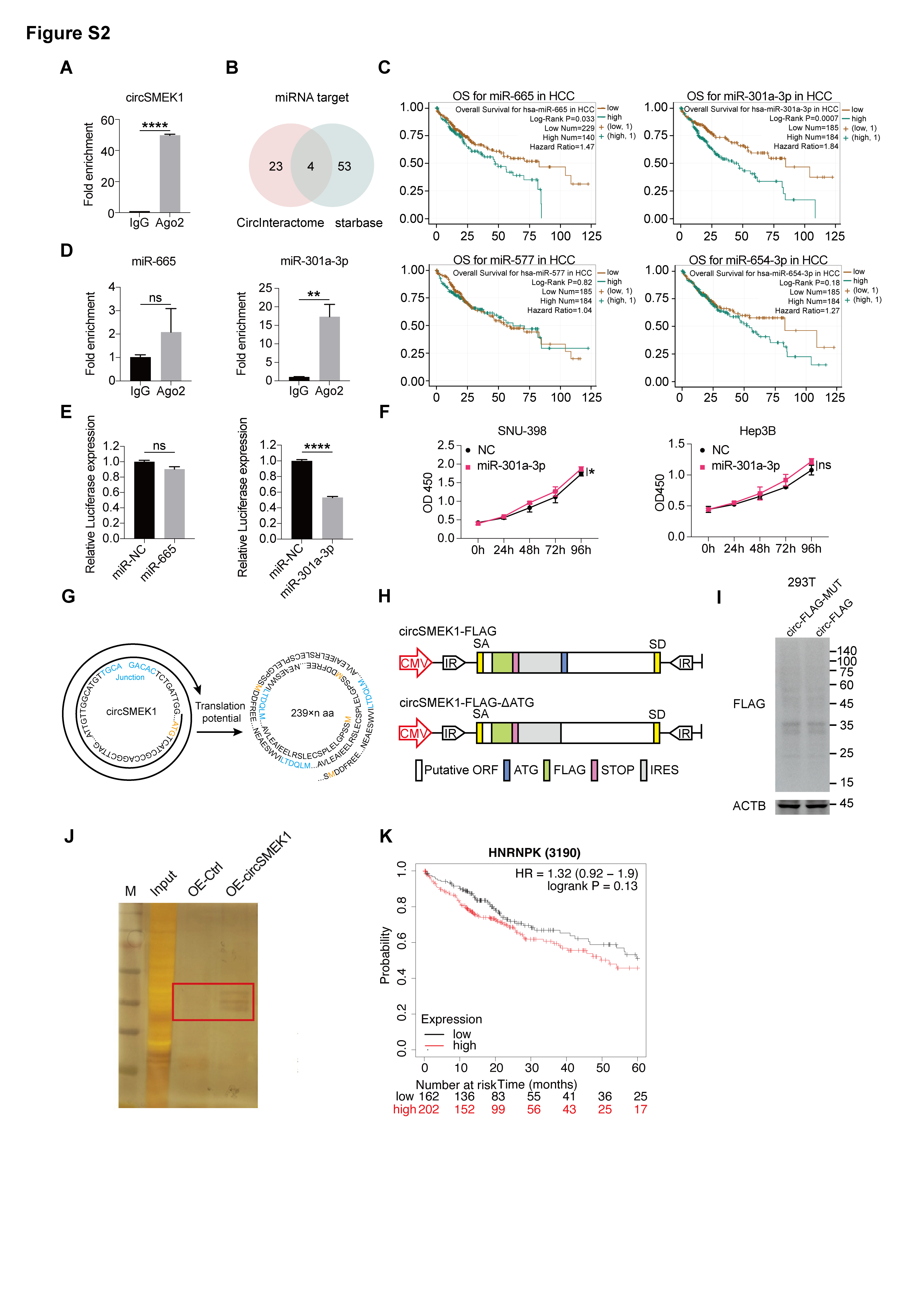


**Figure S2 circSMEK1’s ability as a miRNA-sponge and its translation potential**

**A.** Ago2-RIP demonstrated its ability to be miRNA sponge. **B.** Intersection of potential miRNA target predicted by CircInteractome and starBase. **C.** Overall survival of HCC in TCGA stratified by four miRNA expressions. **D.** Ago2-RIP enrichment of miR-665 and miR-301a-3p. **E.** Luciferase reporter assay showed miR-301a-3p is active while miR-665 is inactive. **F.** CCK-8 assay showed limited function of miR-301a-3p in HCC cells in proliferation. **G.** Schematic diagram illustrating the potential translation model of circSMEK1. **H.** Schematic diagram of plasmid to verify circSMEK1 coding potential. **I.** No specific band was detected after transfected coding-potential verifying plasmid of circSMEK1. **J.** CHIRP assay showed specific bands in 40-55kDa pull-down by biotin-labeled circSMEK1 probes. **J.** Kaplan-Meier analysis showed no significant effect on overall survival in TCGA HCC stratified by *hnRNPK* expression. Data are presented as mean ± SD. P-values were calculated by unpaired two-tailed t-test (A, D, E, F), log-rank test (C), Significant results are presented as *P < 0.05, **P < 0.01, and ***P < 0.001.


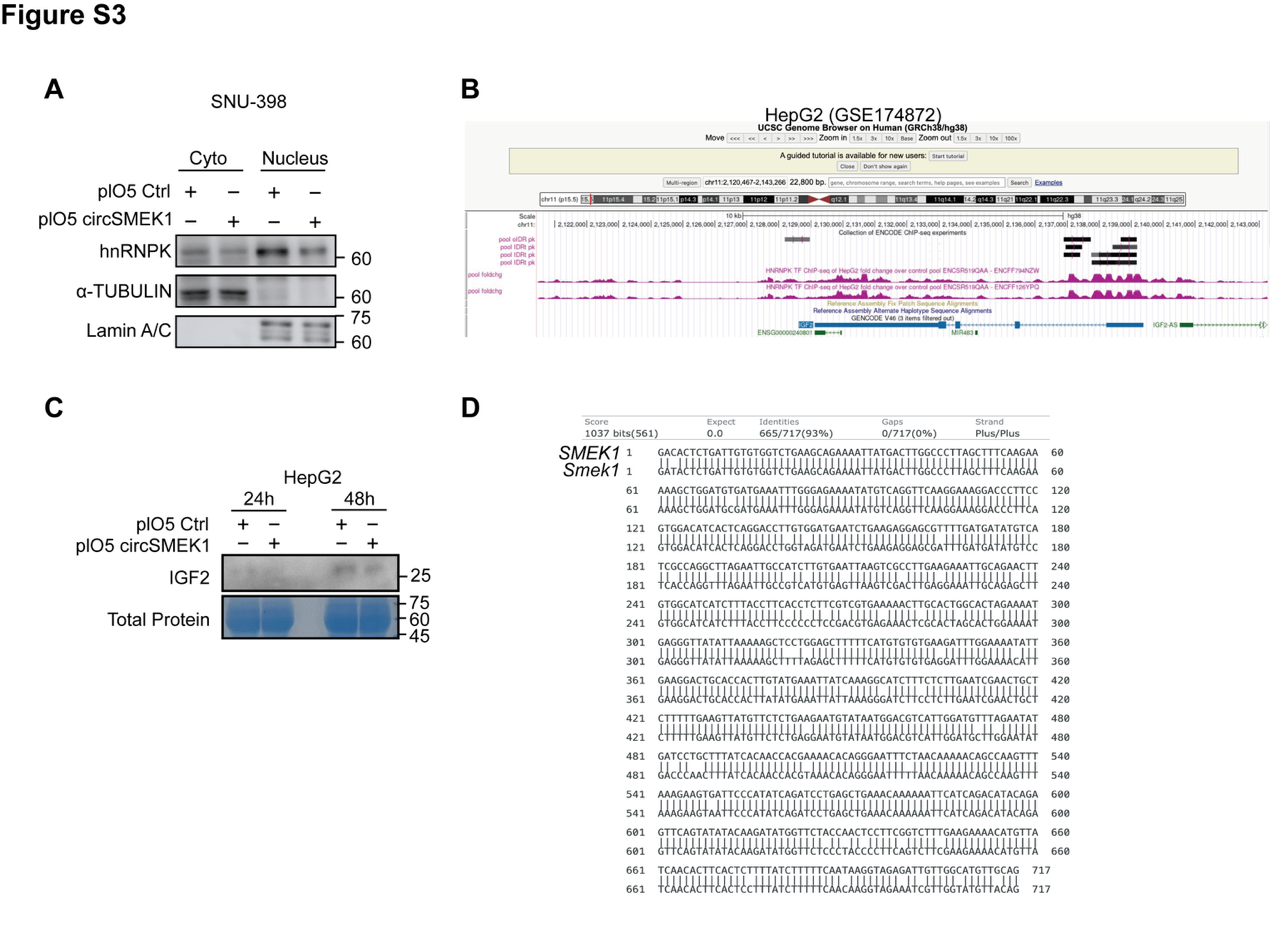


**Figure S3 Characteristics of circSMEK1-hnRNPK-AKT axis**

**A.** Nuclear-cytoplasmic fractionation: OE-circSMEK1 downregulated hnRNPK proteins, which primarily localized in the nucleus. **B.** ChIP-seq analysis for hnRNPK in HepG2 (GSE174872) showed several enrichment peaks at 2000bp upstream of the transcription start site (TSS) of *IGF2.* **C.** Overexpress-circSMEK1 suppresses IGF2 level in 48 h. **D.** Comparison of the sequence of SMEK1 exon4-5 and Smek1


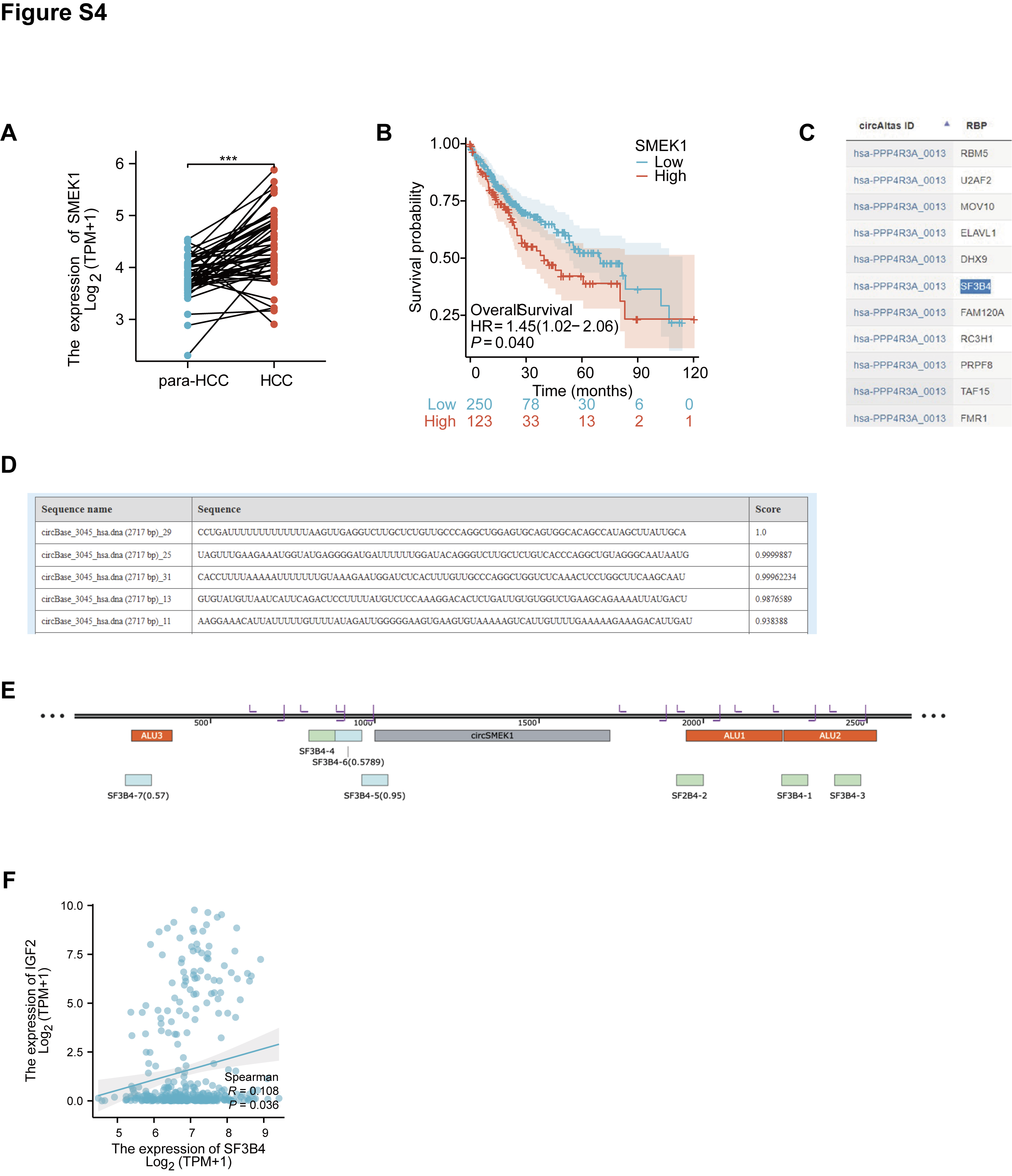


**Figure S4 Characteristics of circSMEK1’s upstream SF3B4**

**A.** The expression of *SMEK1* in paired HCC and para-HCC samples in TCGA. **B.** Kaplan-Meier analysis showed a higher level of *SMEK1* correlated with shorter Overall survival. **C.** Snapshot of CircAtlas of predicted list of RNA binding protein, (January 2024 snapshot) **D.** Sequence of 5 potential binding regions of SF3B4 in the flanking region of circSMEK1 **E.** Schematic diagram of 5 binding sites and circSMEK1 relative location. **F.** Correlation between IGF2 and SF3B4 in TCGA.

**
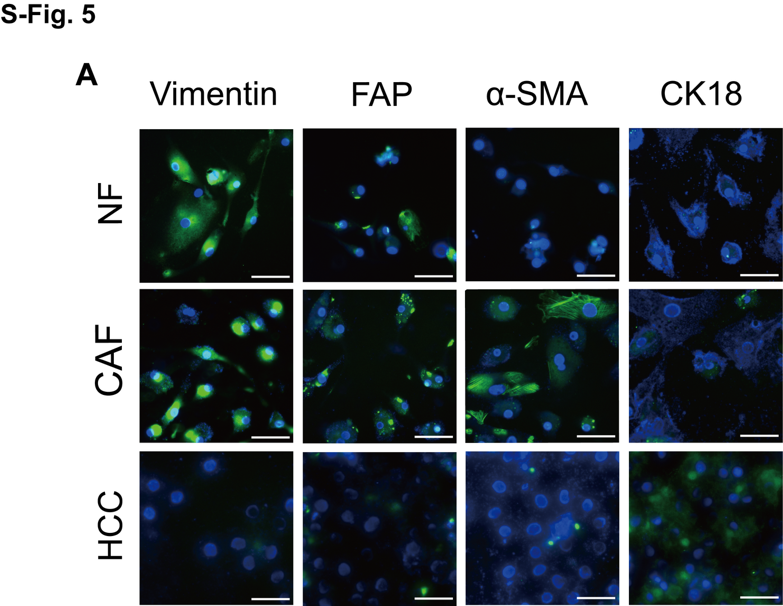
**

**Figure S5 Identification of CAF**

**A.** Identification of NF, CAF and HCC cells by immunofluorescence staining for markers(bar=50μm).

**Additional methods**

**Cell isolation**

For CAFs isolation, we followed the previous publication with adaptations^1^. Tumor tissues were mechanically dissociated and digested with collagenase types 1 and 4 (1.5 mg/mL) at 37°C for 40 min under agitation. After passing cells through a 70μm cell strainer, wash and resuspended cells in Dulbecco's Modified Eagle Medium (DMEM) supplemented with 10% Fetal Bovine Serum (FBS) and 1% penicillin-streptomycin, and plated in culture dishes. After incubation at 37°C for 15 min, the medium with non-adherent cells was removed, leaving behind the adherent cells for culture. Flow cytometry was used to verify that the cells were positive for α-Smooth Muscle Actin (α-SMA) and Fibroblast Activation Protein (FAP) with a purity of over 95%, and negative for Epithelial Cell Adhesion Molecule (EpCAM), Cluster of Differentiation 45 (CD45), or Cytokeratin 18 (KRT18/CK18). All primary cells were used during passages of 1-6. The senescence of the cells was verified at each passage using β-galactosidase staining.

#### NAFLD-like and NASH-like cell model

For NAFLD-like cell model, human liver THLE-2 cell culture for 3 days by DMEM 10% FBS medium with oleic acid at 0.66mM and palmitic acid at 0.33mM**^2^**.

For NASH-like cell model, human liver THLE-2 cell culture for 7 days by DMEM 10% FBS medium with 0.25mM PA, 0.25mM Oleic Acid, 10mM fructose, and 1ug/ml lipopolysaccharide**^3^**.

For Oil red O staining, cells were fixed in 4% paraformaldehyde for 10 min, washed twice with PBS, and pre-treated with 60% isopropanol for 5 min, stained with filtered Oil Red O working solution for 30 min, destained with 60% isopropanol, and finally washed with PBS. Images were captured using a slide scanner (VENTANA DP 200, Roche, Basel, Switzerland). Lipid level is defined as the percentage of Oil Red positive area calculated by Image-Pro plus.

For AST assay, cell culture supernatant was collected and determined by the "aspartic acid substrate method" with Toshiba automatic biochemical analyzer (Beijing North Institute of Biological Technology, Beijing, China). For TG assay, carried out using the Tissue Triglyceride (TG) Content Assay Kit (E1013, Applygen, Beijing, China).

#### CRISPR-RfxCas13d/BSJ-gRNA-Mediated cells

HEK293T cultured in 10 cm dish up to 5×10^6 cells, transfected with 10 μg pLV3-U6-MCS-sgRNA-EF1a-RfxCas13d-Puro, 7.5μg psPAX2, 3μg pMD2.G. Viral supernatant were collected at 48h and 72h, filtrated through 0.45 μm filters. RfxCas13d-expressed stable cell lines were obtained by puromycin selection. NucleofectorTM 2B Device (AAB-1001, Lonza, Cologne, Germany) was used to electroporate plasmids into cells by program T028.

#### Plasmids Construction

#### For circSMEK1 overexpression plasmid, 4-5 exons of *SMEK1* were inserted into the pLO5-ciR vector which was purchased from Geneseed (Guangzhou, China). The backbone vector of pLV was used for constructing hnRNPK, different truncated hnRNPK domains, IGF2, SF3B4.

#### The UB-HA plasmid is from our previous work^4^.

#### For gene knockdown, hairpins of circSMEK1 back-splicing junction, hnRNPK, IGF2, SF3B4 were cloned into plko.1 plasmid; For circRNA knockdown, pLV3-U6-MCS-sgRNA-EF1a-RfxCas13d-Puro was obtained from MiaoLingBio (Hubei, China). 2 sgRNAs targeting the back-splicing junction of circSMEK1 were designed by designing a 26-nt gRNA sequence spanning either side of the BSJ site (13nt + 13nt), (8nt+18nt) following the previous method^5^.

#### For the PiggyBac transposon system, pPB plasmids over-expressing c-MYC, AKT, and NRAS^G12V^ are gifts from Prof. Xuguang Du at China Agricultural University, and pPB plasmids over-expressing circSMEK1 was constructed following previous work^6^ with modifications: pLO5-circSMEK1 plasmid circSMEK1 exon along with forward/backward ~400bp circular frame from pLO5-ciR backbone.

#### RNA extraction

#### For fresh/frozen tissues, serum, or cells, the RNA extraction was performed according to the TRIzol protocol (Ambion, TX, USA).

#### For FFPE tissue, RNA was extracted using the EASYspin kit (Cat. No. RN30, Aidlab, Beijing, China).

#### For subcellular RNA Isolation, after washing with PBS, cells were scraped from 10 cm dishes and pelleted by centrifugation (2,000 rpm, 4°C, 3 min). For cytoplasmic RNA, resuspend pellet in cytoplasmic lysis buffer (10mM Hepes pH7.9, 1.5mM MgCl2, 10mM KCl, 0.1% NP-40, RNase inhibitor) with 3× volume, incubate on ice for 10 min, centrifuge at 5,000 rpm (4°C, 10 min), collect supernatant and wash pellet three times with lysis buffer; For nuclear RNA, resuspend the final pellet in nuclear lysis buffer (50mM Tris-HCl pH7.4, 450mM NaCl, 20% glycerol, 1% NP-40, RNase inhibitor) with 3× volume, vortex vigorously for 30 sec and incubate on ice for 10 min, centrifuge at 12,000 rpm (4°C, 10 min), collect supernatant containing nuclear RNA.

####

#### Reverse transcription Polymerase Chain Reaction (PCR) and quantitative RT-PCR

#### Total RNA was used to synthesize cDNA through HiScript III 1st Strand cDNA Synthesis Kit (R312, Vazyme, Jiangsu, China) with random primer. RT-PCR reaction performed with Hieff® qPCR SYBR Green Master Mix (11201ES, Yeasen, Shanghai, China), on a Lightcycler 480 instrument (Roche, Basel, Switzerland).

**Semi-quantitative RT-PCR Analysis**

Total RNA was extracted from SNU-398 and Hep3B cells using Trizol reagent. For reverse transcription, 0.5 μg of total RNA from each sample was reverse-transcribed into cDNA using a commercial reverse transcription master mix with random hexamer primers according to the manufacturer's instructions, which included simultaneous genomic DNA removal.

To distinguish circSMEK1 from its linear SMEK1 mRNA counterpart, PCR amplification was performed using two distinct primer sets: (1) Convergent primers targeting exons 2-5 of SMEK1 mRNA; (2) Divergent (back-to-back) primers specifically spanning the back-splicing junction of circSMEK1. GAPDH was amplified as an internal control. The resulting PCR products were analyzed by agarose gel electrophoresis, and fragment sizes were determined by comparison with a DNA ladder (Novoprotein, DL2000).

#### RNA Antisense Purification

#### RNA Antisense Purification performed with RAP kit (Bes5103, BersinBio, Guangzhou, China) with modifications following previous work^7^.

**Cell Viability and Colony Formation**

The cell viability was detected using cck-8 regent (5mM WST-8, 0.2mM 1-methoxy PMS, 0.9% NaCl). For colony formation, 1000-1500 cells were seeded into one well of 6-well plates. After 10 days, fixed and stained with 0.1% crystal violet (Sigma, MO, USA) for 10 min, washed twice with PBS.

For Xentuzumab treatment, cells were incubated with 0.5 μM of Xentuzumab.

**RNA Stability Assay**

For the mRNA and circRNA stability assay, cells were treated with 10 μg/mL Actinomycin D (Sigma, MO, USA) to block transcription. After incubation at 0, 2, 6, 12, and 24h, total RNA was collected for qRT-PCR.

**Dual-Luciferase Reporter Assay**

#### The 3′ UTR sequence of IGF2 mRNA was used as the 3′ UTR of firefly luciferase in the vector pGL3. The vector pRL-TK expressing Renilla luciferase was used as a loading control. After transfection in cells for 48 h, cells were lysed, and relative luciferase activities were determined using a Dual-Luciferase Reporter Gene Assay kit (11402ES, Yeasen, Shanghai, China) according to the manufacturer's instructions.

#### RNA immunoprecipitation

#### RNA immunoprecipitation was performed using the PureBinding®RNA Immunoprecipitation Kit (P0101, Geneseed, Guangzhou, China), according to the manufacturer’s protocol.

#### Protein extraction

#### For the routine sample, tissues and cells were lysed in RIPA buffer.

#### For the nucleu-cytoplasmic fraction, cells were isolated by NE-PER™ Nuclear and Cytoplasmic Extraction kit (78833, Invitrogen, CA, USA).

For collecting secreted protein from supernatant of cells, the medium was substituted with DMEM without FBS for 48h, then use the methanol/chloroform method to extract protein. In brief, the medium was collected and centrifuged at 3000 rpm for 5 min at 4 ℃. The supernatant was collected, and methanol of equal volume as well as chloroform of 1/4 volume were added, then fully shaken and mixed for 20s to get a milky suspension. Centrifuge with 12000 rpm for 5 min at 4 ℃, carefully discard the upper water/methanol phase and the lower chloroform phase, and retain the protein in the middle layer. Add 1ml methanol to each sample, shake and mix resolve pipette mixing for resuspension thoroughly, Centrifuge at 12000 rpm for 10 min at 4 ℃, then discard the liquid. 50ul 1x loading buffer was added to resolve pipette mixing for resuspension. From each 6cm-dish-cultured cell, heat at 98 ° C for 5 min to obtain the sample.

#### Western blotting

#### The protein was separated on SDS-PAGE gels and transferred to 0.45 μm PVDF membranes.

For WB of secreted IGF2, the part that over 45kDa of the same piece of WB gel was cut and stained with Coomassie bright blue, the representative band at about 55kDa was selected as the internal reference, and the remaining part of the same piece of gel was transferred to apply antibody development, and the level of secreted IGF2 was detected.

#### Antibodies were used according to the manufacturer’s instructions: hnRNPK (F2371, Selleck, TX, USA), ACTB (AC026, abcloanl, Wuhan, China), IgG (A19711, abcloanl, Wuhan, China), GAPDH (A19056, abcloanl, Wuhan, China), UB (10201, Proteintech, Wuhan, China), α-tubulin(AC007, abcloanl, Wuhan, China), FLAG (20543, Proteintech, Wuhan, China), IGF2 (A2086, abclonal, Wuhan, China), Lamin A/C (A0249, abclonal, Wuhan, China), AKT1 (A17909, abclonal, Wuhan, China), pAKT1 Ser473 (AP0140, abclonal, Wuhan, China), pIGF1R (F0247, Selleck, TX, USA), IGF1R (F0332, Selleck, TX, USA), VEGFA (K112394P, solarbio, Beijing, China), HSP90 (13171, Proteintech, Wuhan, China), SF3B4 (A17608, abclonal, Wuhan, China).

**Immunohistochemistry**

For IHC staining, paraffin-embedded tissue sections were deparaffinized with xylene and rehydrated through a series of decreasing ethanol concentrations. Subsequently, the sections were incubated overnight at 4 °C with the indicated primary antibodies, followed by incubation with the corresponding secondary antibodies at room temperature for 1 hour. After that, Nuclei were stained with hematoxylin. Images were captured using an inverted microscope (Revolve FL, Echo, CA, USA).

The KI67 antibody (A20018, abclonal, Wuhan, China) was used according to the manufacturer’s protocol.

**Immunofluorescence Stainin****g**

#### For IF staining cells were fixed in 4% paraformaldehyde for 10 min after washing with PBS for twice, permeabilized with fresh PBS containing 1% Triton X-100 on ice for 10 min. After blocking with 1% BSA for 30 min at room temperature, samples were incubated with the KI67 (A20018, abclonal, Wuhan, China), FAP (K111339P, solarbio, Beijing, China), α-SMA (F0017, Selleck, TX, USA), CK18 (Ab098863, Aladdin, Shanghai, China), Vimentin (D220268, BBI, Sangon Biotech, Shanghai, China) for 1h at room temperature. With three 5 min washes in PBST buffer, samples were incubated with Secondary Antibody for 1 h at room temperature, protected from light. After three 5 min washes in PBST buffer, nuclei were stained with DAPI, and the images were captured using an inverted microscope (Revolve FL, Echo, CA, USA).

**circSMEK1 Fluorescence in Situ Hybridization (FISH)**

A custom-designed FISH probe targeting circSMEK1 BSJ site with the Cy3-labeled circSMEK1 probe (Genepharma, Shanghai, China). The FISH assay was performed on cells according to the manufacturer’s standard protocol using a commercial FISH kit (Genepharma, Shanghai, China). Images were captured using a fluorescence microscope (Revolve FL, Echo, CA, USA)

**Histological and Pathological Analysis**

For histopathological evaluation, liver tissues and lung tissues (for metastasis assessment) were fixed, paraffin-embedded, and sectioned. Tissue sections (4–5 μm) were stained with hematoxylin and eosin (H&E) according to conventional protocols. Histological changes and metastatic lesions were examined and evaluated by an experienced pathologist. Images were captured using a slide scanner (VENTANA DP 200, Roche, Basel, Switzerland).

**Ubiquitination assays for hnRNPK**

For ubiquitination assays, cells were treated with 20 μM proteasome inhibitor MG132 for 7hr. Determine ubiquitination levels of hnRNPK by immunoblotting with antibody against ubiquitin (10201, Proteintech, Wuhan, China).

**miRNA target prediction**

For miRNA target prediction, by using CircInteractome and starBase, use the intersection part as potential targets.

**Translation prediction**

For circRNA translation prediction, circAtlas 3.0 were used to evaluate translation probability.

**Alphafold3**

The hydrogen bonds between the 3-4 exon of *SMEK1* mRNA and each KH domains of hnRNPK were predicted using AlphaFold 3 (https://alphafoldserver.com/).

**LC-MS/MS**

LC-MS/MS assays were performed at biological mass spectrometry laboratory from the College of Biological Sciences at China Agricultural University as described before^8^. LC-MS analysis using the nano-Acquity nano HPLC (Waters, MA, USA) coupled with a Thermo Q-Exactive high-resolution mass spectrometer (Thermo Scientific, MA, USA).

**Other reagents**

Lipofectamine 3000 (Invitrogen, CA, USA) or PEI (Polysciences, PA, United States) for transfection. Recombinant IGF2 protein was purchased from abclonal (abclonal, Wuhan, China), with a final concentration of 50 ng/ml in experiments. Linsitinib was purchased from Selleck (Selleck, TX, USA), with final concentration of 50 ng/ml in experiments. Xentuzumab was purchased from ApexBio Technology (Apexbio, TX, USA). Spliceostatin A / FR901464 was purchased from Merck (Merck, Darmstadt, Germany). Cycloheximide was purchased from GLPBIO (CHX, GLPBIO, CA, USA), with a final concentration of 100 μg/mL in experiments. MG132 was purchased from Yeasen (Yeasen, Shanghai, China), with a final concentration of 25 μM in experiments.

**References**

1. Song, D., Wu, Y., Li, J., Liu, J., Yi, Z., Wang, X., Sun, J., Li, L., Wu, Q., Chen, Y., et al. (2024). Insulin-like growth factor 2 drives fibroblast-mediated tumor immunoevasion and confers resistance to immunotherapy. J Clin Invest *134*. 10.1172/jci183366.

2. Jin, X., Gao, J., Zheng, R., Yu, M., Ren, Y., Yan, T., Huang, Y., and Li, Y. (2020). Antagonizing circRNA_002581-miR-122-CPEB1 axis alleviates NASH through restoring PTEN-AMPK-mTOR pathway regulated autophagy. Cell Death Dis *11*, 123. 10.1038/s41419-020-2293-7.

3. Jeelani, I., Moon, J.S., da Cunha, F.F., Nasamran, C.A., Jeon, S., Zhang, X., Bandyopadhyay, G.K., Dobaczewska, K., Mikulski, Z., Hosseini, M., et al. (2024). HIF-2α drives hepatic Kupffer cell death and proinflammatory recruited macrophage activation in nonalcoholic steatohepatitis. Sci Transl Med *16*, eadi0284. 10.1126/scitranslmed.adi0284.

4. Jia, X., Liu, H., Ren, X., Li, P., Song, R., Li, X., Guo, Y., and Li, X. (2022). Nucleolar protein NOC4L inhibits tumorigenesis and progression by attenuating SIRT1-mediated p53 deacetylation. Oncogene *41*, 4474-4484. 10.1038/s41388-022-02447-y.

5. Li, S., Wu, H., and Chen, L.-L. (2022). Screening circular RNAs with functional potential using the RfxCas13d/BSJ-gRNA system. Nature Protocols *17*, 2085-2107. 10.1038/s41596-022-00715-5.

6. Xu, C., Qi, X., Du, X., Zou, H., Gao, F., Feng, T., Lu, H., Li, S., An, X., Zhang, L., et al. (2017). piggyBac mediates efficient in vivo CRISPR library screening for tumorigenesis in mice. Proc Natl Acad Sci U S A *114*, 722-727. 10.1073/pnas.1615735114.

7. McHugh, C.A., Chen, C.K., Chow, A., Surka, C.F., Tran, C., McDonel, P., Pandya-Jones, A., Blanco, M., Burghard, C., Moradian, A., et al. (2015). The Xist lncRNA interacts directly with SHARP to silence transcription through HDAC3. Nature *521*, 232-236. 10.1038/nature14443.

8. Song, R., Guo, P., Ren, X., Zhou, L., Li, P., Rahman, N.A., Wołczyński, S., Li, X., Zhang, Y., Liu, M., et al. (2023). A novel polypeptide CAPG-171aa encoded by circCAPG plays a critical role in triple-negative breast cancer. Mol Cancer *22*, 104. 10.1186/s12943-023-01806-x.
